# Fine tuning of the unfolded protein response by ISRIB improves neuronal survival in a model of amyotrophic lateral sclerosis

**DOI:** 10.1038/s41419-020-2601-2

**Published:** 2020-05-26

**Authors:** Ricardo Bugallo, Elías Marlin, Ana Baltanás, Estefanía Toledo, Roberto Ferrero, Rodrigo Vinueza-Gavilanes, Laura Larrea, Montserrat Arrasate, Tomás Aragón

**Affiliations:** 1 Neuroscience Program, Center for Applied Medical Research (CIMA), University of Navarra, 31008 Pamplona, Spain; 2 Gene Therapy and Regulation of Gene Expression Program, Center for Applied Medical Research (CIMA), University of Navarra, 31008 Pamplona, Spain; 3 IDISNA, 31008 Pamplona, Navarra Spain; 4 Department of Preventive Medicine and Public Health, School of Medicine, University of Navarra, 31008 Pamplona, Spain; 5 Centro de Investigación Biomédica en Red Área de Fisiopatología de la Obesidad y la Nutrición (CIBEROBN), 28029 Madrid, Spain

**Keywords:** RNA, Mechanisms of disease, Neuroscience

## Abstract

Loss of protein folding homeostasis features many of the most prevalent neurodegenerative disorders. As coping mechanism to folding stress within the endoplasmic reticulum (ER), the unfolded protein response (UPR) comprises a set of signaling mechanisms that initiate a gene expression program to restore proteostasis, or when stress is chronic or overwhelming promote neuronal death. This fate-defining capacity of the UPR has been proposed to play a key role in amyotrophic lateral sclerosis (ALS). However, the several genetic or pharmacological attempts to explore the therapeutic potential of UPR modulation have produced conflicting observations. In order to establish the precise relationship between UPR signaling and neuronal death in ALS, we have developed a neuronal model where the toxicity of a familial ALS-causing allele (mutant G93A SOD1) and UPR activation can be longitudinally monitored in single neurons over the process of neurodegeneration by automated microscopy. Using fluorescent UPR reporters we established the temporal and causal relationship between UPR and neuronal death by Cox regression models. Pharmacological inhibition of discrete UPR processes allowed us to establish the contribution of PERK (PKR-like ER kinase) and IRE1 (inositol-requiring enzyme-1) mechanisms to neuronal fate. Importantly, inhibition of PERK signaling with its downstream inhibitor ISRIB, but not with the direct PERK kinase inhibitor GSK2606414, significantly enhanced the survival of G93A SOD1-expressing neurons. Characterization of the inhibitory properties of both drugs under ER stress revealed that in neurons (but not in glial cells) ISRIB overruled only part of the translational program imposed by PERK, relieving the general inhibition of translation, but maintaining the privileged translation of ATF4 (activating transcription factor 4) messenger RNA. Surprisingly, the fine-tuning of the PERK output in G93A SOD1-expressing neurons led to a reduction of IRE1-dependent signaling. Together, our findings identify ISRIB-mediated translational reprogramming as a new potential ALS therapy.

## Introduction

Loss of protein folding homeostasis has been identified as a common pathological feature of most neurodegenerative diseases (NDs), including Parkinson’s, Alzheimer’s disease, or amyotrophic lateral sclerosis (ALS), a fatal neurodegenerative disorder caused by degeneration of upper and lower motor neurons. Among the protein quality control mechanisms that serve to restore proteostasis in neurons, the unfolded protein response (UPR) has been recently proposed as a key determinant of neuronal fate in NDs^1–3^.

Three independent UPR mechanisms initiate a transcriptional response to restore proteostasis, and remodel translation to (1) reduce the load of proteins to be folded in the ER, and (2) enhance the translation of a select subset of stress-regulated mRNAs^4^. The translational arm of the UPR is regulated by the ER stress sensor PERK (PKR-like ER kinase) via eIF2 α-subunit (eIF2α) phosphorylation. eIF2α phosphorylation (p-eIF2α) prevents the recycling of the eIF2 complex, thereby repressing translation initiation of most cellular messenger RNAs (mRNAs). As a relevant exception to this regulatory mechanism, a subset of mRNAs bearing small upstream open reading frames (uORFs) at their 5′-untranslated regions is translationally privileged under eIF2-limiting conditions. Among them, translation of the mRNA encoding the transcription factor ATF4 (activating transcription factor 4) facilitates the expression of antioxidant and amino acid import genes^5–8^. The versatile translational/transcriptional program established by PERK is also initiated by three other eIF2α kinases, PKR, HRI, and GCN2, that are activated either by viral infections, and heme or amino acid deprivation, respectively. By providing a common output to distinct stresses, this pathway is known as the integrated stress response (ISR)^9–11^.

Parallel to PERK, two independent mechanisms initiated by IRE1 (inositol-requiring enzyme-1) and ATF6 (activating transcription factor 6) complete the cellular response to ER stress. IRE1 is an ER-resident kinase/endonuclease; upon activation, IRE1 excises a non-conventional 26-nucleotide intron within the mRNA encoding the XBP1 protein^12–14^, which, in turn, facilitates the translation of spliced XBP1 protein (XBP1s), a potent UPR transcription factor^15^.

Beyond its neuroprotective roles, under chronic or unresolvable stresses, PERK and IRE1 can initiate a pro-apoptotic signaling and promote neurodegeneration^1,3^. This dual, fate-defining capacity of the UPR is most relevant in post-mitotic cells, playing a prominent role in several NDs.

UPR activation has been extensively documented in ALS. Most ALS patients suffer sporadic forms of the disease and only 5–10% of cases have a family history of the disease (fALS)^16,17^. Mutations in *SOD1* gene account for ~20% of fALS cases^18^. Other fALS-inducing mutations include a nucleotide sequence (G4C2) expansion within the *C9orf72* gene^19,20^, or mutations in RNA-binding proteins like TDP43 or FUS^21,22^. UPR activation has been documented in the spinal cord of sporadic ALS patients^23–26^, as well as in motor neurons derived from fALS patient-iPS cells^27–29^. In fALS mouse models, presymptomatic UPR activation was identified in the most vulnerable, fast fatigable motor neurons as an early pathological event^30–32^.

To establish the role of UPR signaling in ALS progression, genetic and pharmacological modulation of specific UPR components in mouse disease models has been attempted. Most of these efforts were focused on the exacerbation or suppression of PERK signaling. For instance, pharmacological enhancement of PERK via Salubrinal^30^, Guanabenz^33,34^, or Sephin1^35^ has been proposed to delay/improve disease progression in mutant SOD1 mouse models. Accordingly, genetic inhibition of eIF2α phosphatase GADD34^36^ improved ALS symptomatology. In line with a neuroprotective effect of PERK signaling, ALS was aggravated by crippling PERK expression^37^. Nevertheless, these evidences remain controversial. A recent publication claims that genetic inhibition of PERK signaling pathway in mutant SOD1-based models does not ameliorate ALS symptoms^38^. Also, the capacity of Guanabenz to improve disease progression in SOD1-based models has been questioned^39^. Supporting the notion that inhibition, not exacerbation of PERK signaling, may be therapeutically relevant the genetic ablation of ATF4 delayed disease progression in a SOD1-based model^40^. These conflicting evidences have also been observed in TDP43-based ALS models where both enhancement and suppression of PERK signaling prevent neurodegeneration^41,42^.

This controversy exists partly because the temporal and causal relationship between UPR activation and neuronal death has not been precisely quantified. Quantifying these relationships has the potential to identify the contribution of specific UPR mechanisms to neuronal survival, leading to the development of better therapeutic strategies. Here, we have recapitulated the toxicity of SOD1 fALS alleles in a neuronal model where neuronal death was documented by longitudinal survival analysis. This methodology allows us to quantitatively score the risk of neuronal death associated to predictive factors (i.e., UPR levels) by Cox regression models^43–46^. Using UPR fluorescent reporters, we have longitudinally monitored the amplitude and dynamics of UPR, and confirmed that UPR preceded and was associated with neuronal death. Most importantly, pharmacological targeting of specific UPR processes led us to identify a novel therapeutic strategy for ALS that mitigates ER stress and enhances neuronal survival.

## Materials and methods

### Plasmids

Plasmids pCAGGs-mCherry (Ch) and pCAGGs-GFP (green fluorescent protein) were already described^45^. SOD1 versions (WT/G85R/G93A) from pcDNA3.1 SOD1 plasmids (Addgene, Watertown, MA) were subcloned into pCAGGS empty vector (pCAGGs-SOD1WT, pCAGGs-G85RSOD1, pCAGGs-G93ASOD1). pCAGGs-SOD1Ch tagged version was generated in two steps. First, an *Age*I site was inserted in pCAGGS-SOD1WT plasmid by PCR with primers Fiii forward: GACGGCTGCCTTCGGGGG and *Xho*I*Age*I reverse: GTACCTCGAGCGTTAACCGGTTTGGGCGATCCCAATTACACCACAAG.

Next, the Ch coding sequence from pTAP242 was inserted in the *Age*I site. pCAGGs-G85RSOD1Ch and pCAGGs-G93ASOD1Ch were generated by inserting SOD1 G85R and G93A from pCAGGS-SOD1 plasmids (G85R and G93A, respectively) into pCAGGS-SOD1WTCh plasmid by digestion with *Bsa*I and *Psp*OMI.

pGL3-5XUPRE-GFP was generated in two steps. First, pGL3-5XUPRE-Venus was created by replacing the firefly luciferase ORF in pGL3-5XUPRE-Luc (courtesy of Dr. Ron Prywes) with a PCR product containing the Venus coding sequence (from pCAGGS-ERAI-Venus) bearing *Nco*I and *Xba*I sites at their 5′ and 3′ ends, respectively (forward: ATATCCATGGTGAGCAAGGGCGAGGAGCTG; reverse: TATTTCTAGATTTACTTGTACAGCTCGTCCATGCCG).

Next, the Venus coding sequence in pGL3-5XUPRE-Venus was replaced by the GFP ORF (from pCAGGS-SynGFP plasmid^45^) by *Nco*I/*Bsr*GI digestion generating the pGL3-5XUPRE-GFP plasmid.

To generate pGL3-GFP, the DNA encoding the luciferase ORF in pGL3-Luc (Promega) plasmid was replaced by a DNA fragment encoding the enhanced GFP (eGFP) coding using restriction enzymes *Xba*I and *Nco*I.

The pCAGGs-SpR (splicing reporter) plasmid was constructed by fusing a 1.47-kb murine XBP1 gene fragment that contains the XBP1 UPR intron to eGFP (Clontech Laboratories, Mountain View, CA) and inserted into a pCAGGS vector at the *Eco*RI and *Nhe*I restriction sites.

The sequence of all plasmids was verified by Sanger sequencing.

### Neuronal and cell cultures

Animal handling was carried out in accordance with the European Community Council Directive (2010/63/EC) and Spanish legislation (Real Decreto 53/2013); ethical protocols were approved by the Ethics Committee of the University of Navarra (051-13 and 038-18). Primary cultures of cortical neurons were established from E20 Sprague–Dawley rat embryos as described elsewhere^45^. Both HEK293 (ATCC) and HeLa (ATCC) cells were maintained in 5% CO_2_ at 37 °C, Dulbecco’s modified Eagle’s medium (DMEM) (Gibco, Thermo Fisher Scientific, Waltham, MA) supplemented with 10% fetal bovine serum (FBS), 1% sodium pyruvate (Gibco), and 1% penicillin–streptomycin (Gibco). HEK293 and HeLa cells were regularly tested for mycoplasma contamination.

### Pharmacological modulation of UPR mechanisms

The ER stress pharmacological inducers tunicamycin (Tun) (Applichem) and thapsigargin (Thap) (Applichem), as well as the UPR modulators ISRIB (Sigma), 4μ8C (Sigma), GSK2606414 (GSK) (Toronto Research Chemicals, North York, Canada), and Sephin1 (Sigma), were dissolved in dimethyl sulfoxide (DMSO) (Sigma) and stored at −80 °C.

For survival and UPR activation experiments, neuronal cultures were supplemented with a single dose of ISRIB (0.1, 0.5, or 1 μM), 4μ8C (5 or 16 µM), or GSK (0.5, 2, 4, or 8 µM), and the highest equivalent dose of DMSO as a control 1 day post transfection.

To characterize UPR activation, HEK293 cells and primary cortical neurons were treated with Thap (100 nM) alone or in combination with the UPR modulators described above [ISRIB (500 nM), GSK (2 µM), or 4µ8C (5, 16, and 50 µM), and Sephin1 (10 µM), and DMSO as a control]. Cells were harvested for Western blot (WB) analysis at different time points after treatment (from 1.5, 3, 6, 18 to 24 h).

### Lipofectamine transfection of primary rat cortical neurons

Primary cortical neurons were transfected with Lipofectamine 2000 transfection reagent (Invitrogen, Carlsbad, CA) after 5 days in vitro (DIV5). One hour before adding the DNA mixture, the culture medium was replaced by Neurobasal medium (Gibco). Transfection was conducted following the manufacturer’s protocol. Depending on the experiment, 1–3 μg of DNA were transfected per 24-well plate well. Two–three hours after transfection, transfected wells were washed and the transfection medium was replaced by Neurobasal medium supplemented with 1% FBS, 1% GlutaMAX (Gibco), 2% B27 (Gibco), 0.1 mg/ml gentamicin (Life Technologies, Carlsbad, CA) and 2.5 mg/ml fungizone (Gibco). Usually, for longitudinal survival experiments 4 wells per condition and 2 plates (replicates) per independent experiment were transfected.

### Calcium phosphate transfection of HEK293 cells

HEK293 cells were transfected using calcium phosphate method. Briefly, cells were plated in 6-well plates as described above the day before transfection in order to reach a 50–70% confluence the day of transfection. One hour before adding the DNA-containing calcium phosphate precipitate, the culture medium was replaced by DMEM without FBS. Four micrograms of the corresponding plasmid were used in each transfection. In co-transfection experiments, 3 μg of each plasmid were used per well. Cells were incubated with the transfection mix for 2–5 h at 37 °C, and once the calcium phosphate precipitate was visible as a thin granulate in the culture, the medium was replaced by 2 ml of DMEM + 10% FBS per well.

### Western Blot analysis

Whole-cell lysates from rat primary cortical neurons or confluent HEK293 cells were harvested for Western blot analysis. Cell cultures monolayers were washed twice with ice-cold phosphate-buffered saline (PBS). Next, cells were scraped in 250 μl of Laemmli buffer [50mM Tris pH 6.8 (Bio-Rad, Hercules, CA), 100 mM dithiothreitol (Sigma), 2% sodium dodecyl sulfate (SDS) (Bio-Rad), 10% glycerol (Sigma), 0.01% bromophenol blue (Merck, Darmstadt, Germany), Phos-STOP phosphatase inhibitor cocktail (Roche, Basel, Switzerland), 17.5 mM β-glycerol phosphate (Sigma), and Complete protease inhibitor cocktail (Roche)]. Extracts were boiled at 95 °C for 10 min.

Protein samples were loaded onto polyacrylamide Bis-Tris gels (Invitrogen) (% of polyacrylamide of the gel varied from 7% up to 12%) and separated by electrophoresis at room temperature (RT) at a constant voltage of 100 V for over 2.5 h in SDS running buffer (0.1% SDS, 25 mM Tris, 192 mM glycine). The proteins were then transferred to nitrocellulose membranes (Bio-Rad) for 1 h at 350 mA and 4 °C in transfer buffer [20% methanol (Applichem, Barcelona, Spain), 192mM glycine (Bio-Rad), and 25 mM Tris (Bio-Rad)]. The membranes were incubated for 40 min at RT with blocking solution (5% non-fat milk and/or 5% bovine serum albumin (Sigma) in TBST-buffer (20 mM Tris-HCl (pH 8.0), 150 mM NaCl, 0.05% Tween-20 (Sigma)). After washing, membranes were probed overnight at 4 °C with specific primary antibodies (see below) diluted in blocking solution. Membranes were washed four times for 10 min with TBST and incubated for 2 h at RT with the specific secondary antibodies (see below) diluted in blocking solution. Finally, membranes were washed three times for 10 min with TBST and once for 10 min with TBS. Electrochemiluminescence (ECL) reactions were performed with Lumigen ECL Ultra (Lumigen, Southfield, MI) reagent or Western Lightning Plus-ECL (PerkinElmer, Waltham, MA) and visualized in Odyssey Fc (LI-COR, Lincoln, NE). Bands were quantified with the Image Studio 5.2 software.

### Immunofluorescence

For immunofluorescence experiments, neuronal cultures were fixed 2 days after transfection, unless stated otherwise. Neuronal medium was replaced by 4% paraformaldehyde (PFA)–4% sucrose (Fluka, Thermo Fisher Scientific, Waltham, MA) dissolved in PBS. Eight minutes after PFA fixation, neurons were washed twice with PBS. Neurons were kept at 4 °C until immunostaining. The immunofluorescence protocol details have been already described^45^. Images were obtained with a ×63 objective on a Zeiss Axiovert 200M Fluorescence microscope (Zeiss, Oberkochen, Germany), and their acquisition and processing was performed using MetaMorph Microscopy Automation and Image Analysis Software (Molecular Devices, San Jose, CA). At least *n* = 15 neurons were required for each condition on each independent experiment.

### Detection of newly synthesized proteins

Rat primary cortical neurons or HEK293 cells were first treated with different compounds (DMSO, Thap, ISRIB, and GSK). Five hours after drug addition, the growth media were replaced by methionine-free DMEM (Gibco). Then, to label nascent proteins, cells were incubated with 50 µM l-homopropargylglycine (HPG) at 37 °C, 5% CO_2_ for 30 min. After this incubation, cells were washed with PBS (Gibco), followed by fixation with 3.7% formaldehyde in PBS at RT for 15 min. Immunolabeling after HPG incorporation was performed with Alexa Fluor 488 using reagents and following protocols from Click-iT™ HPG Alexa Fluor™ 488 Protein Synthesis Assay Kit (Thermo Fisher).

### Automated image acquisition

Longitudinal survival experiments were performed in rat primary cortical neurons growing in 24-well plates, using the automated microscope Zeiss Observer Z1, equipped with a chamber that maintains both temperature and CO_2_ levels stable: 37 °C and 5% CO_2_, as previously described^45^. For a typical survival experiment, 15 non-overlapping positions per well and 4 wells per condition were used. Positions were chosen randomly, making the selection of neurons to analyze unbiased. A template with the same initial spatial positions was used until the experiment finished, following exactly the same neuronal fields. For survival experiments, images were acquired with the ×10 objective. In experiments where UPR activation was monitored longitudinally, 75–150 images per condition were acquired with the ×20 objective at manually selected positions.

### Image processing and statistics

Fluorescence intensity was quantified in single neurons and HEK293 cells with the Metamorph Analysis software. The final average fluorescence intensity values were the result of subtracting the background signal from the fluorescence intensity measured in an area corresponding to the neuronal nucleus or cytoplasm, depending on the labeled protein of interest. When measuring fluorescence intensities from multiple channels, the same selected areas were transferred among the images corresponding to each channel. GraphPad Prism 5 software (GraphPad Software, San Diego, CA) was used to perform correlation tests, Mann–Whitney, Kruskal–Wallis, and Dunn’s multiple comparison tests, two-way analysis of variance (ANOVA), and Bonferroni multiple comparisons. Normal distribution of data was assessed in all cases by Shapiro–Wilk test. Data not following a normal distribution were analyzed with non-parametric statistics. GraphPad Prism 5 software was also used to obtain the graphs. For survival experiments, Matlab-based (The Matworks, Natick, MA) semi-automated ad hoc programs were developed for image analysis and to estimate the survival times of individual neurons as described previously^45^. The program shows the same neuronal field sequentially at all different experimental times. At the initial time, the neurons were selected and numbered. In the rest of the time series, the software opens each image and the user compares it with the previous time. Dead neurons are identified and categorized as censored events. Neurons that survive until the end of the experiment are considered uncensored events. The data were exported to Excel and all further survival analyses were performed with STATA 12 (StataCorp, College Station, TX). Codes are available upon request.

The Nelson–Aalen cumulative hazard function was used to plot the differences in the cumulative risk of death among the experimental groups. At least *n* = 100 neurons was required for each condition on each independent experiment. These differences were analyzed with clustered Cox regression models, as long as a proportional hazard assumption was fulfilled (Shoenfeld residual-based test evaluation or graphical assessment). Since transfection conditions are not controlled at the level of the individual neuron, a clustered Cox regression analysis of neurons co-existing in the same well was performed to improve the accuracy of the test. In addition, and because each independent experiment may include replicates (one or two plates), the variability in the baseline toxicity between experiments was adjusted by stratifying the Cox model for each plate.

To longitudinally analyze UPR activation in single neurons, we designed a specific Matlab-based custom software based on particle tracking applications^45^. The user selects each neuron at the initial time and the program automatically detects those neurons at subsequent times, measuring the fluorescence intensity in the red channel. To this end, the image is binarized by applying a threshold and several morphological operations are performed in order to determine the pixels that correspond to each neuron. The centroid of the neuron at a given time is then obtained and used to look for the presence of the neuron (in the same region) at the following time point. This is possible due to the fact that neurons remain roughly at the same positions throughout the experiment. Additionally, the pixels corresponding to each neuron (obtained from the red channel) are used to calculate the average green fluorescence in the neuron (green channel) corresponding to the UPR fluorescent reporter. Automated analysis enabled blinded assessing of the outcome. Codes are available upon request. GraphPad Prism 5 software was used to analyze the data (two-way ANOVA and Bonferroni’s post hoc tests) and to obtain the graphs.

To assess if 5XUPRE expression was associated with longer neuronal survival, we longitudinally monitored 5XUPRE fluorescence levels every 24 h for each single neuron, and ascertained survival time for all neurons. Cox regression analysis was used to determine the association between 5XUPRE expression and neuronal survival. We categorized 5XUPRE expression into quartiles, according to the distribution of 5XUPRE expression at the different follow-up times. We assessed if 5XUPRE expression was associated with neuronal death. As a second step, we adjusted the Cox regression models for neuronal group.

### Antibodies

- SOD1:
  • pan-SOD1: Calbiochem/Merck (574597). IF (1:150)
  • C4F6: Medimabs (Montreal, Canada) (MM-0070-2). IF (1:100)
  • 8B10: ThermoScientific (MA1-105). IF (1:1000)
- mCherry: Abcam (Cambridge, UK) (Ab125096). WB (1:2000), IF (1:500)
- GFP: Life Technologies (Carlsbad, CA) (A11122). WB (1:1000), IF (1:200)
- PERK: Cell Signaling (Danvers, MA) (#3192). WB (1:1000)
- p-PERK (Thr980): Cell Signaling (#3179S). WB (1:1000)
- ATF4: Cell Signaling (#11815). WB (1:1000), IF (1:100)
- p-eIF2α (Ser51)ː Cell Signaling (#9721). WB (1:1000)
- Total eIF2αː Santa Cruz (Dallas, TX) (sc-11386). WB (1:1000)
- CHOP: Cell Signaling (#2895). WB (1:1000)
- XBP1s: Cell Signaling (#12782). WB (1:1000)
- Heme oxygenase 1 (HO-1): Enzo Life Sciences (Farmingdale, NY) (ADI-SPA-895-D). WB (1:1000), IF (1:200)
- MAP2: Sigma (M1406-.2ML). IF (1:1000)
- GFAP: Sigma (G3893-.2ML). IF (1:1000)
- GAPDH: Cell Signaling (#2118). WB (1:1000)
- Tubulin: Sigma (T6199). WB (1:10,000)
- ECL anti-mouse IgG HRP linked: GE Healthcare (Chicago, IL) (NA931). WB (1:15,000)
- ECL anti-rabbit IgG HRP linked: GE Healthcare (NA934). WB (1:10,000)
- FITC-conjugated anti-mouse IgG: Jackson Immunoresearch (Ely, UK) (115-095-003). IF (1:200)
- Cy5-conjugated anti-mouse IgG: Jackson Immunoresearch (115-175-166). IF (1:500)
- Cy3-conjugated anti-mouse IgG: Jackson Immunoresearch (115-165-003). IF (1:500)
- Cy5-conjugated anti-rabbit IgG: Jackson Immunoresearch (115-165-144). IF (1:500)
- Alexa 488-conjugated anti-rabbit IgG: Invitrogen (A-21206). IF (1:500)

## Results

### A neuronal ALS model where mutant SOD1 toxicity is quantitated by longitudinal survival analysis

**Fig. 1:**
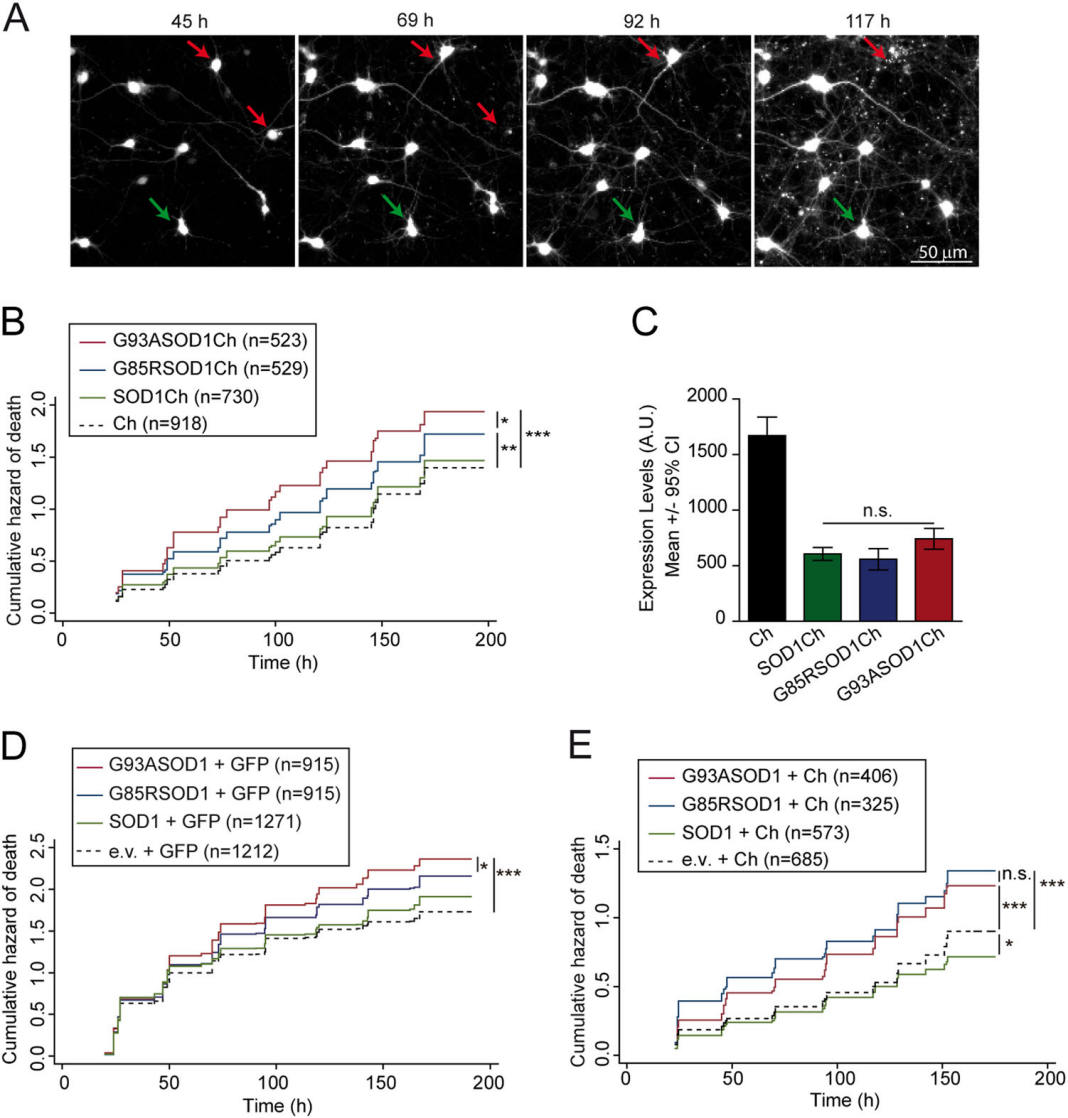
Pathological SOD1 mutants G93A and G85R increase significantly the risk of neuronal death in rat cortical primary neurons. **a** Example of longitudinal tracking of individual primary cortical neurons expressing monomeric Cherry (Ch) with automated microscopy. Red arrows indicate neurons that die over the course of the experiment. Green arrows point to a neuron tracked longitudinally that survive up to 117 h. **b** Cumulative hazard estimates of primary neurons transfected with WT and pathological versions of SOD1 (SOD1Ch, G85RSOD1Ch, G93ASOD1Ch) and Ch as control. Cox proportional hazard (CPH) analysis; pooled data from three independent experiments. **c** Ch fluorescence intensity of individual neurons 20–24 h after transfection (Kruskal–Wallis and Dunn’s post hoc test, Ch = 66, SOD1Ch = 60, G85RSOD1Ch024 and G93ASOD1Ch = 54 neurons from a representative experiment). **d** Cumulative hazard estimates of primary neurons co-transfected with GFP and SOD1 versions (WT and pathological mutants G85R and G93A). CPH analysis; pooled data from four independent experiments. **e** Cumulative hazard estimates of primary neurons co-transfected with Ch and SOD1 versions. CPH analysis, pooled data from two independent experiments. Number of neurons (*n*), all error bars indicate 95% confidence intervals (CIs), non-significant (n.s.), *p < 0.05, **p < 0.01, and ***p < 0.001.

First, we developed a neuronal model to score the toxicity of pathogenic SOD1 mutant proteins by longitudinal survival analysis^45,47–50^. Rat primary cortical neurons were transfected with plasmids encoding wild-type (WT) (SOD1Ch) or ALS mutant SOD1 (G85RSOD1Ch, G93ASOD1Ch) alleles expressed as fusion proteins with a C-terminal monomeric Cherry fluorescent protein (Ch) tag; as a control, a plasmid encoding non-toxic protein Ch was transfected. Fluorescent protein tagging enabled the identification and tracking of SOD1-expresing neurons by automated microscopy (Fig. 1a, Supplementary Fig. S1), allowing us to estimate the survival time of individual neurons (last time each neuron was observed alive) as well as SOD1 protein levels (Supplementary Fig. S2A, S2B). Statistics for longitudinal survival analysis were applied for data analysis. Using Cox proportional hazard (CPH) analysis, differences in survival were estimated relative to a reference group (Ch) and expressed as hazard ratio (HR). Mutant SOD1 versions (G85R and G93A) increased significantly the risk of death (HR coefficients relative to Ch: SOD1Ch, HR 1.01, *P* = 0.808, 95% confidence interval (CI) 0.88–1.16; G85RSOD1Ch, HR 1.25, *P* < 0.01, 95% CI 1.08–1.46; G93ASOD1Ch, HR 1.48, *P* < 0.001, 95% CI 1.32–1.68) (Fig. 1b). Since the expression levels of all SOD1 variants were similar (Fig. 1c), differences in survival result from the intrinsic toxicity/conformation that fALS mutations convey to SOD1.

As a formal caveat, fluorescent epitope tagging may affect SOD1 folding or toxicity; however, analysis of SOD1 folding with conformational and pan-SOD1 antibodies did not reveal significant differences between Ch-tagged and untagged SOD1 versions (Supplementary Fig. S2C, S2D). The toxicity of untagged SOD1 proteins was also scored by co-transfection of neurons with plasmids expressing untagged SOD1 versions and GFP or Ch constructs. Taking advantage of the high co-transfection efficiency achieved (Supplementary Fig. S2E), GFP or Ch fluorescence was used to identify and track those neurons expressing untagged SOD1 proteins, which displayed similar toxicities as their Ch-tagged counterparts (Fig. 1d, e).

### Longitudinal analysis establishes a link between mutant SOD1-dependent neuronal death and UPR activation

**Fig. 2:**
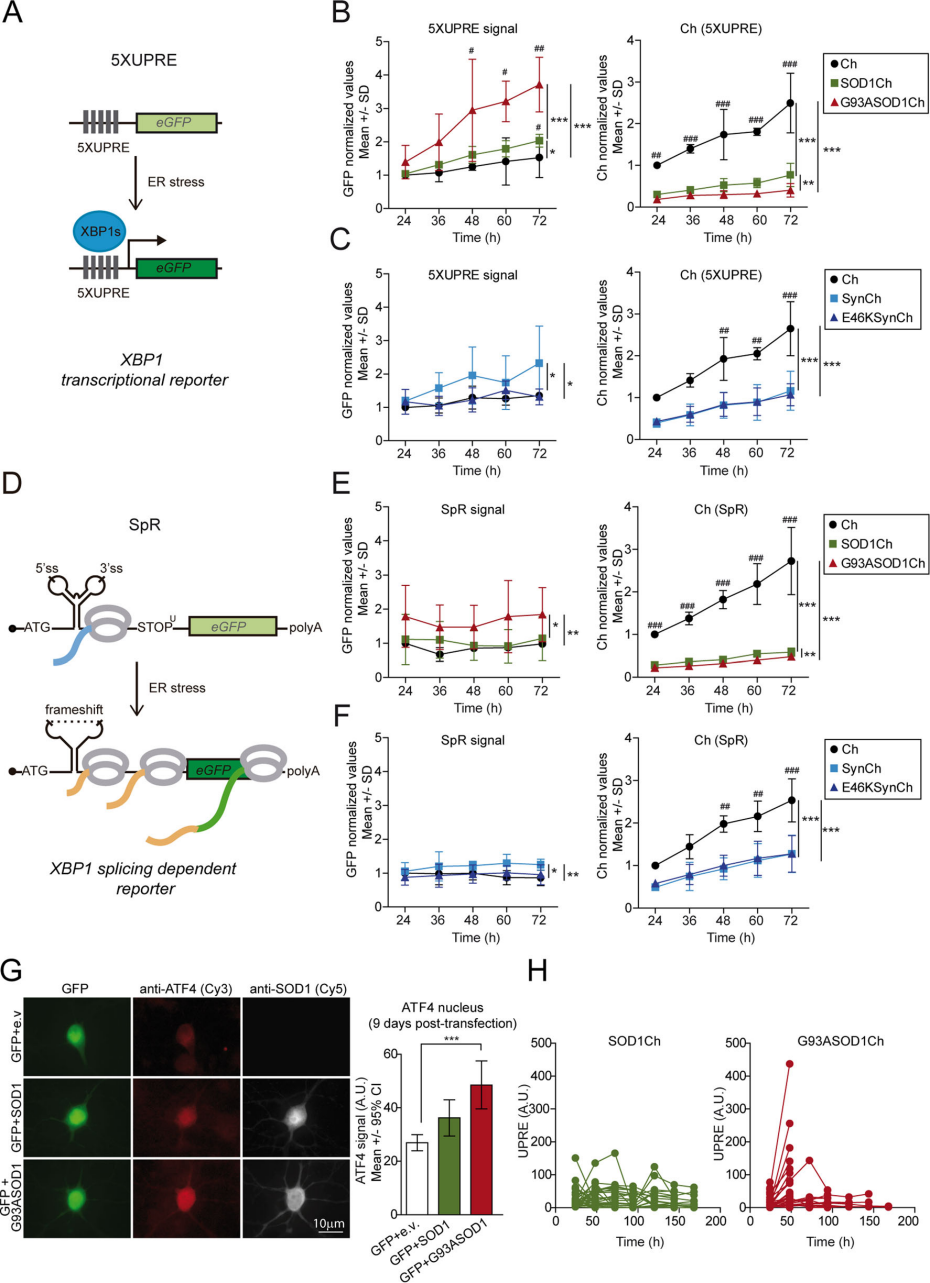
UPR signaling is activated in G93A SOD1-expressing neurons. **a** Scheme of the GFP-based transcriptional reporter 5XUPRE. Upon ER stress the XBP1 spliced transcription factor (XBP1s) binds five tandem repeats of the UPR element (5XUPRE), thereby enabling GFP transcription. **b** Left graph: GFP levels quantification over time measured in individual neurons expressing Ch or Ch-tagged versions (SOD1Ch, G93ASOD1Ch) and the transcriptional reporter 5XUPRE. GFP levels were normalized against control condition (Ch, 24 h post transfection). Two-way ANOVA and Bonferroni pairwise comparisons; 5XUPRE levels change significantly among Ch-tagged versions (*F*_2,30_ = 22.3, *p* < 0.001) and with time (*F*_4,30_ = 6.6, *p* < 0.001). Right graph: Ch fluorescence levels quantification (surrogate of protein levels) from neurons analyzed on the left graph. Protein levels change significantly among Ch-tagged versions (*F*_2,30_ = 119.8, *p* < 0.001) and with time (*F*_4,30_ = 9.3, *p* < 0.001). There is significant interaction in the treatments with time (*F*_8,30_ = 2.7, *p*_int_ < 0.05). **c** Left graph: GFP levels quantification over time measured in individual neurons expressing Ch or Ch-tagged versions (SynCh, E46KSynCh) and the transcriptional reporter 5XUPRE. GFP levels were normalized against control condition (Ch, 24 h post transfection). Two-way ANOVA and Bonferroni pairwise comparisons; 5XUPRE levels change significantly among Ch-tagged versions (*F*_2,30_ = 6.1, *p* < 0.01). Right graph: Ch fluorescence levels quantification from neurons analyzed on the left graph. Protein levels change significantly among Ch-tagged versions (*F*_2,30_ = 50.9, *p* < 0.001) and with time (*F*_4,30_ = 13.1, *p* < 0.001). **d** Scheme of GFP-based XBP1-splicing-dependent reporter (SpR). Under ER stress, splicing of the 26-nucleotide XBP1 intron provides the translational frameshift that allows GFP translation. **e** Left graph: GFP levels quantification over time measured in individual neurons expressing Ch or Ch-tagged versions (SOD1Ch, G93ASOD1Ch) and the transcriptional reporter SpR. GFP levels were normalized against control condition (Ch, 24 h post transfection). Two-way ANOVA and Bonferroni pairwise comparisons; SpR levels change significantly among Ch-tagged versions (*F*_2,30_ = 7.7, *p* < 0.01). Right graph: Ch fluorescence levels quantification from neurons analyzed on the left graph. Protein levels change significantly among Ch-tagged versions (*F*_2,30_ = 164.2, *p* < 0.001) and with time (*F*_4,30_ = 12.9, *p* < 0.001). There is significant interaction in the treatments with time (*F*_8,30_ = 5, *p*_int_ < 0.001). **f** Left graph: GFP levels quantification over time measured in individual neurons expressing Ch or Ch-tagged versions (SynCh, E46KSynCh) and the transcriptional reporter SpR. GFP levels were normalized against control condition (Ch, 24 h post transfection). Two-way ANOVA and Bonferroni pairwise comparisons; SpR levels change significantly among Ch-tagged versions (*F*_2,30_ = 5.2, *p* < 0.05). Right graph: Ch fluorescence levels quantification from neurons analyzed on the left graph. Protein levels change significantly among Ch-tagged versions (*F*_2,30_ = 39.5, *p* < 0.001) and with time (*F*_4,30_ = 14.4, *p* < 0.001). **b**, **c**, **e**, **f** Data from *n* = 3 experiments; 100–300 neurons per time point, for each condition, in each individual experiment; SD, standard deviation. Significant differences of treatments: ***p < 0.001, **p < 0.01, and *p < 0.05. Significant differences among time: ^###^*p* < 0.001, ^##^*p* < 0.01, and ^#^*p* < 0.05. Values come from individual neurons but not from the same neurons along the experimental time. **g** Immunofluorescence analysis of ATF4 and SOD1 expression (8B10) in primary cortical neurons co-transfected with plasmids expressing GFP and SOD1 (WT/G93A)-untagged versions or empty vector (e.v.) as control. Cy3 and Cy5 staining documents ATF4 and SOD1 levels, respectively. Quantification of ATF4 nuclear levels in individual neurons expressing GFP and e.v., WT SOD1, and mutant G93A SOD1 at 9 days post transfection (number of neurons per condition; GFP + e.v. = 25, GFP + SOD1 = 24, GFP + G93ASOD1 = 25; representative experiment of three independent experiments). Kruskal–Wallis and Dunn's post hoc test. **h** Individual primary neurons co-transfected with 5XUPRE transcriptional reporter and C-terminally tagged SOD1 (WT/G93A) forms were monitored longitudinally over time. The graph shows 5XUPRE levels and survival time for each individual neuron (36 neurons per condition). All error bars indicate 95% confidence intervals (CI), *p < 0.05, **p < 0.01, and ***p < 0.001.

Next, we characterized longitudinally the amplitude and kinetics of UPR activation of neurons expressing WT or mutant SOD1 using two kinds of UPR fluorescent reporters: (1) a transcriptional UPR reporter, 5XUPRE, that bears five tandem copies of the UPR element (5XUPRE) (Fig. 2a)^51,52^, and (2) a splicing reporter, SpR, based on the non-canonical splicing of XBP1^53^ (Fig. 2d). The capacity of both reporters to monitor ER stress was validated both in HEK293 cells and primary neurons treated with chemical ER stress inducers Thap or Tun (Supplementary Fig. S3). As a control, UPR activation was also documented in neurons expressing Ch protein.

Since UPR activation has been observed in a wide variety of neurodegenerative disorders, it may be considered as a general neuronal death mechanism. If that was the case, UPR should be turned on in response to different neurotoxic proteins. To test this possibility, we analyzed UPR activation in neurons overexpressing WT α-synuclein (aSyn), or the pathological E46K aSyn mutant allele, that we recently identified as highly neurotoxic^45^. Of note, both E46K aSyn and G93A SOD1 convey similar risks of death to neurons in our experimental model. Primary neurons were first co-transfected with plasmids expressing SOD1 (SOD1Ch, G93ASOD1Ch) or aSyn (SynCh, E46KSynCh) and the 5XUPRE reporter. A significant increase in 5XUPRE-derived fluorescence was observed in G93A SOD1 over WT SOD1 or control Ch-expressing neurons (Fig. 2b). Interestingly, WT aSyn enhanced 5XUPRE activation (to a lower extent than G93A SOD1) but the E46K aSyn mutant did not, indicating that UPR activation is not linked to neurotoxicity in a general, unspecific manner (Fig. 2c). A similar trend was observed with the SpR reporter. Mutant SOD1 increased significantly SpR activation (Fig. 2e). Again, WT aSyn slightly increased SpR fluorescence, while E46K aSyn yielded background levels of SpR fluorescence (Fig. 2f). These observations recapitulated the specific activation of UPR by mutant SOD1 described previously; in spite of its high toxicity, E46K aSyn mutant failed to trigger UPR.

In our hands, fluorescent indicators of PERK activation were not sensitive enough to document the activation of this UPR branch in neurons. To overcome this limitation, we analyzed nuclear ATF4 protein levels in neurons expressing WT or G93A SOD1 by immunofluorescence. We first validated this method in Thap-treated primary cultures where a robust increase in nuclear ATF4 levels was observed in neurons, and, to a lesser extent, in non-neuronal cell types (astrocytes) (Supplementary Fig. S4A, S4B). Expression of G93A SOD1 induced a modest but significant increase in nuclear ATF4 levels (Fig. 2g, Supplementary Fig. S4C). The capacity of mutant SOD1 to induce ATF4 expression was also observed by WB in mutant SOD1-expressing HEK293 cells (Supplementary Fig. S4D). Thus, our neuronal model is able to recapitulate the activation of IRE1 and PERK signaling by G93A SOD1.

The longitudinal inspection of UPR reporter dynamics in individual cells led us to observe that UPR activation preceded death in a major fraction of neurons expressing G93ASOD1Ch (Fig. 2h). This trend was not observed in neurons expressing WT SOD1Ch, and suggested a direct involvement of ER stress/UPR signaling in neuronal fate. To determine if UPR activation was associated with neuronal death, neurons co-transfected with G93ASOD1Ch and 5XUPRE expression plasmids (Ch as control) were subjected to longitudinal survival analysis. Cox regression analysis was performed to address if the levels of UPR fluorescence at early time points could predict neuronal death. Neurons expressing higher 5XUPRE activity levels (quartiles 3 and 4 vs. quartile 1) at early time points of the time course (24, 48, and 72 h) were associated with a higher neuronal death risk (Table 1). This association remained statistically significant for 5XUPRE expression at 24 h after adjustment by group (Table 2). Taken together, these results demonstrate that UPR activation is positively associated with neuronal death.

**Table 1:** CPH model of the effect of 5XUPRE levels on neuronal survival.

	24 h	48 h	72 h	96 h
*n*	523	405	365	329
HR Q1 (ref.)	1	1	1	1
HR Q2 (95% CI)	1.29, *p* = 0.125 (0.93–1.79)	1.24, *p* = 0.291 (0.82–1.88)	1.32, *p* = 0.229 (0.83–2.10)	1.39, *p* = 0.198 (0.78–2.14)
HR Q3 (95% CI)	1.48, *p* < 0.05* (1.07–2.04)	1.56, *p* < 0.05* (1.05–2.32)	1.64, *p* < 0.05* (1.05–2.55)	1.51, *p* = 0.107 (0.91–2.49)
HR Q4 (95% CI)	1.82, *p* < 0.001*** (1.33–2.5)	1.59, *p* < 0.05* (1.07–2.37)	1.58, *p* < 0.05* (1.00–2.49)	1.20, *p* = 0.471 (0.72–2.02)

*n* number of neurons, *HR* hazard ratio, *Q1, Q2, Q3, Q4* quartiles of 5XUPRE levels, *95% CI* 95% confidence interval, *h* hours, *ref* reference group. *p < 0.05, ***p < 0.001.

**Table 2:** CPH model of the effect of 5XUPRE levels on neuronal survival adjusted by groups (Ch and G93ASOD1Ch).

	24 h	48 h	72 h	96 h
*n*	523	405	365	329
HR Q1 (ref.)	1	1	1	1
HR Q2 (95% CI)	1.26, *p* = 0.162 (0.91–1.75)	1.26, *p* = 0.261 (0.84–1.90)	1.27, *p* = 0.310 (0.80–2.01)	1.30, *p* = 0.308 (0.78–2.14)
HR Q3 (95% CI)	1.55, *p* < 0.01** (1.12–2.14)	1.41, *p* = 0.090 (0.94–2.09)	1.62, *p* < 0.05* (1.03–2.53)	1.40, *p* = 0.194 (0.84–2.30)
HR Q4 (95% CI)	1.81, *p* < 0.001*** (1.32–2.48)	1.40, *p* = 0.099 (0.93–2.09)	1.32, *p* = 0.231 (0.83–2.08)	0.98, *p* = 0.956 (0.58–1.70)
HR Ch (ref.)	1	1	1	1
G93ASOD1Ch (95% CI)	2.99, *p* < 0.001*** (2.26–3.96)	2.83, *p* < 0.001*** (2.03–3.95)	2.89, *p* < 0.001*** (2.01–4.15)	2.94, *p* < 0.001*** (1.97–4.40)

*n* number of neurons, *HR* hazard ratio, *Q1, Q2, Q3, Q4* quartiles of 5XUPRE levels, *95% CI* 95% confidence interval, *h* hours, *ref* reference group. *p < 0.05, **p < 0.01, ***p < 0.001.

### ISRIB decreases G93A SOD1-dependent neuronal death

**Fig. 3:**
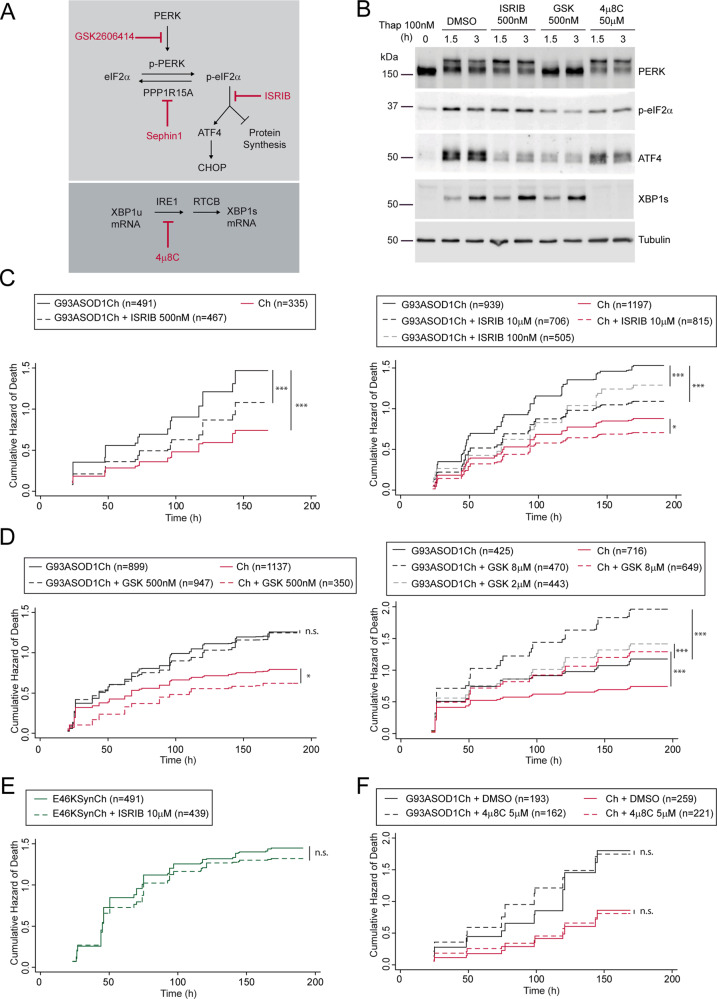
ISRIB decreases G93A SOD1-dependent neuronal death. **a** Scheme depicting the modes of action of GSK2606414 (GSK), ISRIB, Sephin1, and 4μ8C in PERK and IRE1-dependent signaling. XBP1u (unspliced XBP1), XBP1s (spliced XBP1), and RTCB (RNA ligase enzyme that joins the exons resulting from XBP1 intron removal by IRE1). **b** Western blot analysis of PERK, p-eIF2α, ATF4, XBP1s, and tubulin (loading control) from HEK293 protein extracts treated with 100 nM thapsigargin (Thap) and 500 nM ISRIB, 500 nM GSK, 50 μM 4μ8C, and DMSO as control. Representative experiment from at least *n* = 3. **c** Cumulative hazard estimates of primary neurons transfected with SOD1 pathological version G93ASOD1Ch and treated with 500 nM, 100 nM, and 10 μM ISRIB (Ch as control). CPH analysis; pooled data from three independent experiments (left panel) and five independent experiments (right panel). **d** Cumulative hazard estimates of primary neurons transfected with G93ASOD1Ch and treated with 500 nM, and 2 and 8 μM GSK (Ch as control). CPH analysis; pooled data from three independent experiments (left panel) and two independent experiments (right panel). **e** Cumulative hazard estimates of primary neurons transfected with aSyn pathological version (E46KSynCh) and treated with 10 μM ISRIB. CPH analysis; pooled data from three independent experiments. **f** Cumulative hazard estimates of primary neurons transfected with G93ASOD1Ch and treated with 5 μM 4μ8C (Ch as control). CPH analysis; representative experiment from two independent experiments. Number of neurons (n), non-significant (n.s.), *p < 0.05 and ***p < 0.001.

IRE1 and PERK signaling mechanisms encode the dual capacity to promote neuronal death or survival. Therefore, we assessed whether the pharmacological modulation of these two UPR signaling branches could affect G93A SOD1-dependent neurodegeneration. Specifically, we used GSK (a direct inhibitor of PERK kinase activity^54^), ISRIB (a downstream PERK inhibitor that reverses the effects of p-eIF2α^55^), and 4μ8C (which inhibits IRE1 endonuclease domain^56^). The mode of action of these drugs is summarized in Fig. 3a.

ISRIB, GSK, and 4μ8C displayed the anticipated inhibitory roles in Thap-treated HEK293 cells (Fig. 3b, Supplementary Fig. S5). ER stress-induced PERK activation results in its *trans*-autophosphorylation^55^, which is reflected in a characteristic electrophoretic shift, and the increased p-eIF2α that, in turn, stimulates the translation of uORF-containing transcripts (i.e., ATF4 mRNA). As anticipated, GSK blocked PERK and p-eIF2α, and the ensuing ATF4 translation. Acting as a downstream PERK inhibitor, ISRIB blocked ATF4 synthesis by acting downstream of p-eIF2α. As a bona fide IRE1 RNAse inhibitor, 4μ8C strongly impaired XBP1 splicing and prevented XBP1s synthesis without affecting PERK signaling significantly. Of note, inhibition of either UPR branch did not seem to perturb the other one.

Next, we tested their ability to modulate neuronal survival by longitudinal survival analysis in neurons expressing Ch-tagged G93A SOD1 or Ch (as control). Importantly, ISRIB enhanced the survival of G93A SOD1-expressing neurons within a wide dose range between 100nM and 10 μM (Fig. 3c). This neuroprotective effect was selective because ISRIB did not improve the survival of E46K aSyn expressing neurons (Fig. 3e). Since ISRIB modulates PERK-dependent translational regulation, we anticipated that GSK might display the same protective effect as ISRIB. Surprisingly, GSK did not improve the survival of G93A SOD1-expressing neurons; if any, it decreased neuronal survival at the higher doses (Fig. 3d).

PERK signaling can be enhanced through the inhibition of the p-eIF2α phosphatase, GADD34/PPP1R15A. Pharmacological inhibition of PPP1R15A with Sephin1 has been proposed to alleviate ALS symptoms in mutant SOD1 transgenic mice^35^. When tested in HeLa cells, Sephin1 stimulated ATF4 synthesis in the absence of stress. Surprisingly, under ER stress, Sephin1 did not sustain high ATF4 protein levels, but strongly inhibited XBP1 expression, consistently with previous observations^57^ (Supplementary Fig. S6A). In our hands, Sephin1 did not improve neuronal survival (Supplementary Fig. S6B). Finally, inhibition of IRE1-dependent XBP1 splicing with 4μ8C did not affect survival of G93A SOD1-expressing neurons (Fig. 3f).

These evidences indicated that ISRIB was the only UPR modulator tested that was able to improve the survival of G93A SOD1-expressing neurons.

### ISRIB tunes neuronal PERK signaling

**Fig. 4:**
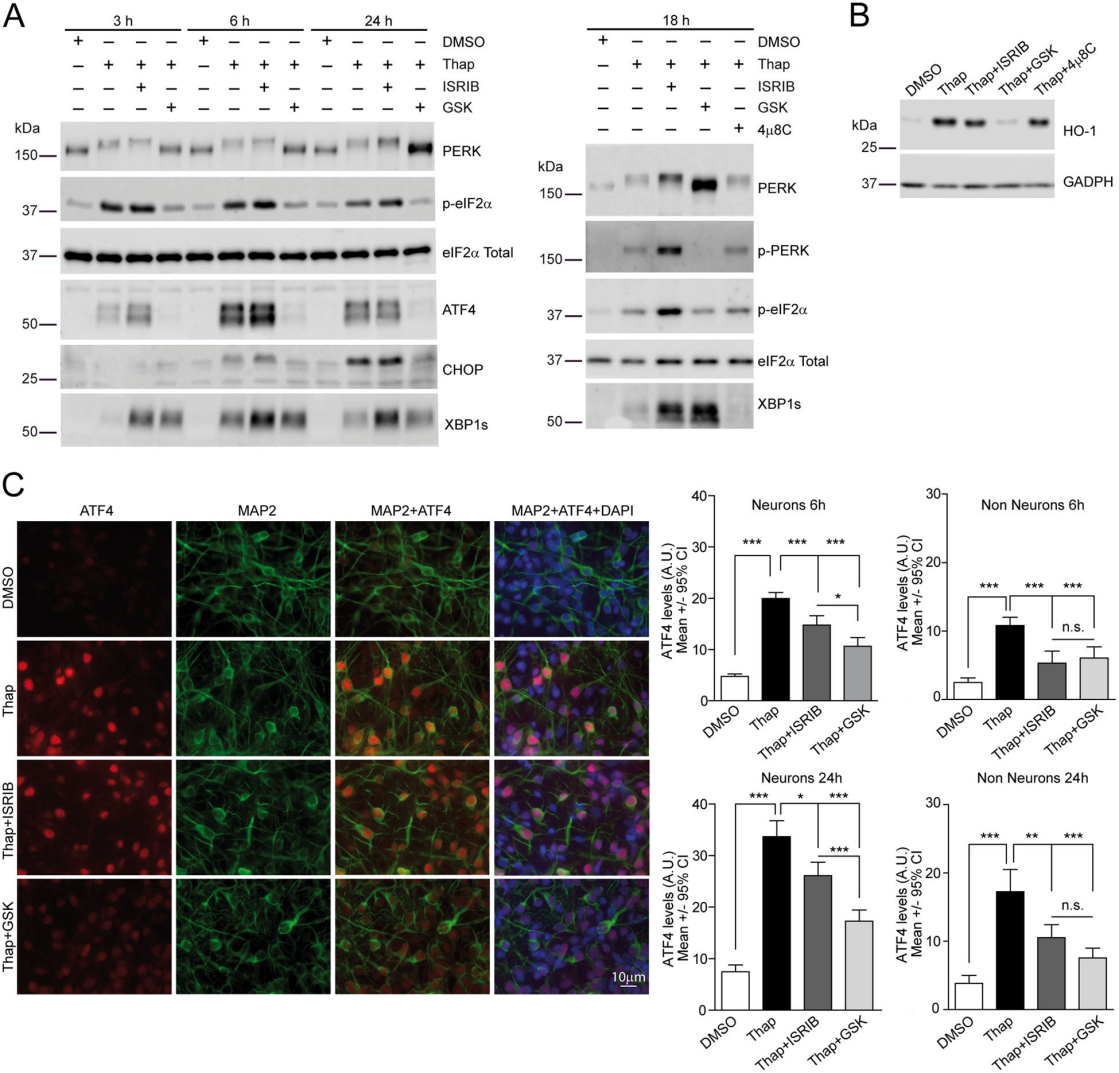
ISRIB and GSK2606414 (GSK) modulate neuronal PERK signaling differently under acute ER stress. **a** PERK and IRE1 signaling was analyzed by WB in protein extracts from primary neurons treated with DMSO (control) or 100 nM Thap either alone or in combination with 500 nM ISRIB, 2 μM GSK, and 16 μM 4μ8C at different time points. Representative experiment from *n* = 3. **b** HO-1 protein levels were analyzed by WB in protein extracts from primary neuronal cultures treated with DMSO (control) or 100 nM Thap either alone or in combination with 500 nM ISRIB, 2 μM GSK, or 16 μM 4μ8C. Representative experiment from *n* = 3. **c** Representative images of ATF4 and MAP2 immunofluorescence staining in primary neuronal cultures after 6 h treatment with 100 nM Thap and 500 nM ISRIB or 2 μM GSK. Graphs showing quantification of nuclear ATF4 levels in neurons (MAP2+) and non-neuronal cells (MAP2−) after 6 and 24 h of treatment with Thap, ISRIB, and GSK (number of neurons per condition 6h: DMSO = 84, Thap = 87, Thap + ISRIB = 78, Thap + GSK = 67; number of neurons per condition 24 h: DMSO = 54, Thap = 73, Thap + ISRIB = 78, Thap + GSK = 57; number of non-neuronal cells per condition 6 h: DMSO = 52, Thap = 37, Thap + ISRIB = 36, Thap + GSK = 50; number of non-neuronal cells per condition 24 h: DMSO = 49, Thap = 28, Thap + ISRIB = 54, Thap + GSK = 50; representative experiment from three independent experiments. Kruskal–Wallis and Dunn’s post hoc test. All error bars indicate 95% confidence intervals (CI), (n.s.) non-significant, *p < 0.05, **p < 0.01, and ***p < 0.001.

The different capacity of ISRIB and GSK to modulate mutant SOD1-dependent neuronal death prompted us to further evaluate the mode of action of these drugs in primary neuronal cultures under ER stress conditions. Surprisingly, we observed that, unlike in HEK293 cells, ISRIB did not reduce ATF4 expression in Thap-treated neuronal cultures, while GSK efficiently blunted ATF4 expression. Similarly, translation of CHOP mRNA (another uORF-regulated transcript) was suppressed by GSK, but not by ISRIB (Fig. 4a). As a consequence of the differential modulation of ATF4 by ISRIB and GSK, ISRIB was unable to inhibit transcription of ATF4 target genes, such as the antioxidant heme oxygenase 1 (*HO-1*) gene^58^ (Fig. 4b). Interestingly, modulation of PERK with either GSK or ISRIB resulted in the enhanced expression of XBP1s protein (Fig. 4a), which was not observed in HEK293 cells. As anticipated, IRE1 inhibition by 4μ8C decreased XBP1s protein levels in primary neurons (Fig. 4a).

To determine if neurons, and no other cell types represented in the primary neuronal cultures, were resistant to ISRIB action, we analyzed by immunofluorescence nuclear ATF4 protein levels in neuronal (MAP2+) versus non-neuronal (MAP2−) cells, treated as described above. In line with the previous WB analyses, ISRIB slightly reduced ATF4 nuclear levels in neurons, while GSK efficiently dampened nuclear ATF4 in this cell type. Interestingly, ISRIB diminished ATF4 levels as efficiently as GSK in non-neuronal cells (Fig. 4c).

**Fig. 5:**
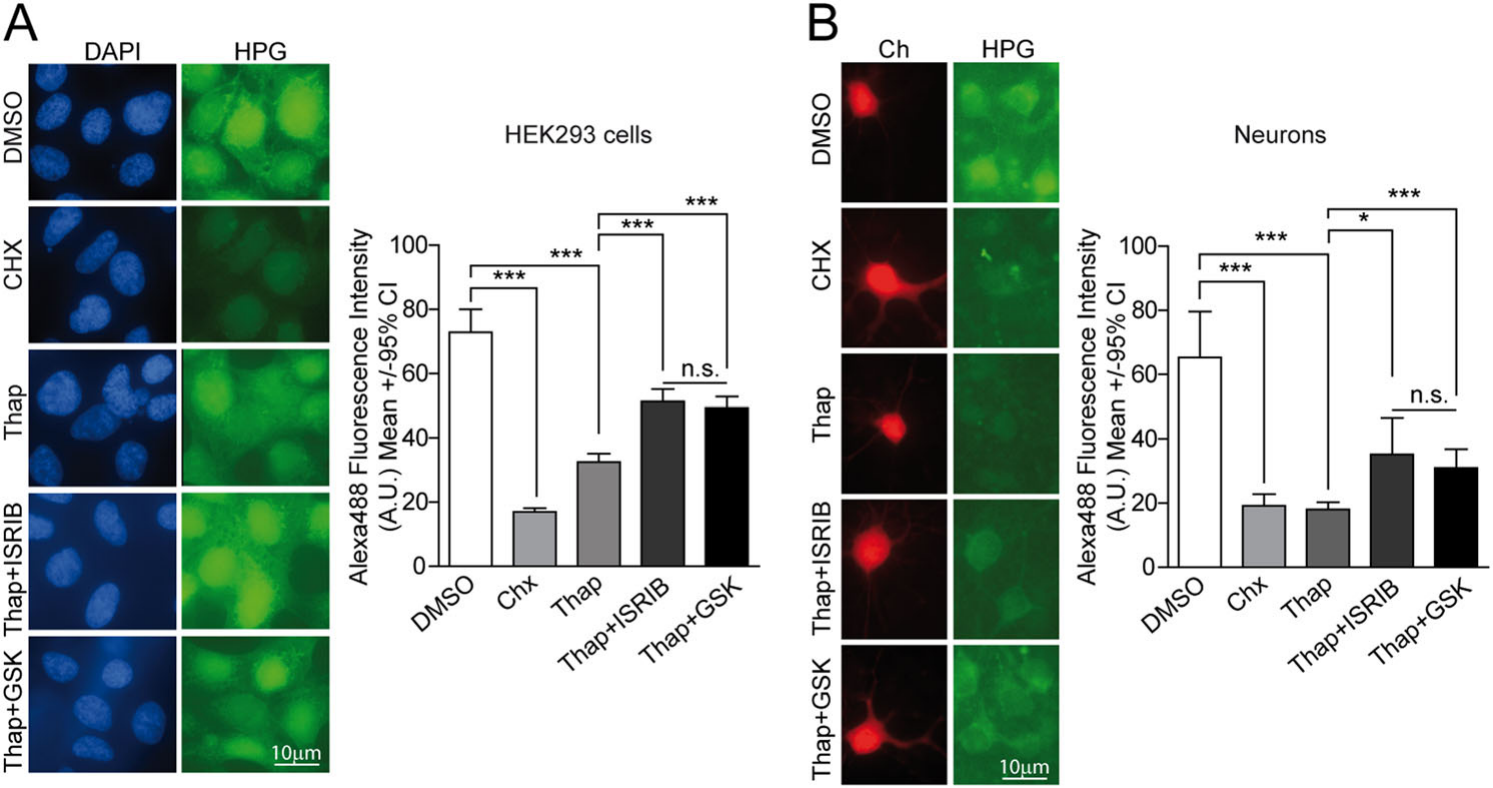
ISRIB and GSK relieve PERK-mediated translational repression with similar efficiencies. **a** l-Homopropargylglycine (HPG) pulse experiments were performed to measure translation rates of HEK293 cells treated for 5 h with 100 nM Thap either alone or in combination with 500 nM ISRIB, and 2 μM GSK or DMSO (control). Representative images of HPG incorporation (Alexa 488 fluorescence) and fluorescence quantification of individual cells. Representative experiment from two independent experiments; number of cells per condition: DMSO = 164, Chx = 160, Thap = 132, Thap + ISRIB = 154, and Thap + GSK = 163. **b** HPG metabolic labeling were performed in Ch-transfected neurons as described in **a**. Representative images of HPG incorporation (Alexa 488 fluorescence) and fluorescence quantification in treated individual neurons. Number of neurons per condition: DMSO = 39, Chx = 40, Thap = 39, Thap + ISRIB = 40, and Thap + GSK = 40 from two independent experiments, Kruskal–Wallis and Dunn’s post hoc test. All error bars indicate 95% confidence intervals (CI), (n.s.) non-significant, *p < 0.05, and ***p < 0.001.

As we confirmed that ISRIB could not efficiently suppress uORF-dependent translation, we tested if it could relieve the translational shutdown imposed by p-eIF2α. To quantify protein synthesis rates in neurons (but not in glial cells), we measured the incorporation of a methionine analog HPG, containing an alkyne moiety^59^, where fluorophores can be coupled by click chemistry. HPG pulse-chase experiments served to quantify translation of individual cells/neurons. To confirm that these measurements faithfully documented protein translation, a control treatment with the translational inhibitor cycloheximide was included. As anticipated, Thap-mediated p-eIF2α significantly decreased protein synthesis rates in neurons. To our surprise, ISRIB and GSK recovered protein translation with similar efficiencies both in HEK293 cells and in neurons, where ISRIB/GSK-mediated recovery was less efficient (Fig. 5).

Altogether, these evidences indicate that in response to ER stress, ISRIB selectively recovers protein translation rates in neurons, but fails to suppress translation of uORF-containing mRNAs, such as ATF4 and CHOP. Therefore, ISRIB provides a fine-tuning of the translational program established by PERK.

### ISRIB decreases G93A SOD1-dependent ER stress signaling

**Fig. 6:**
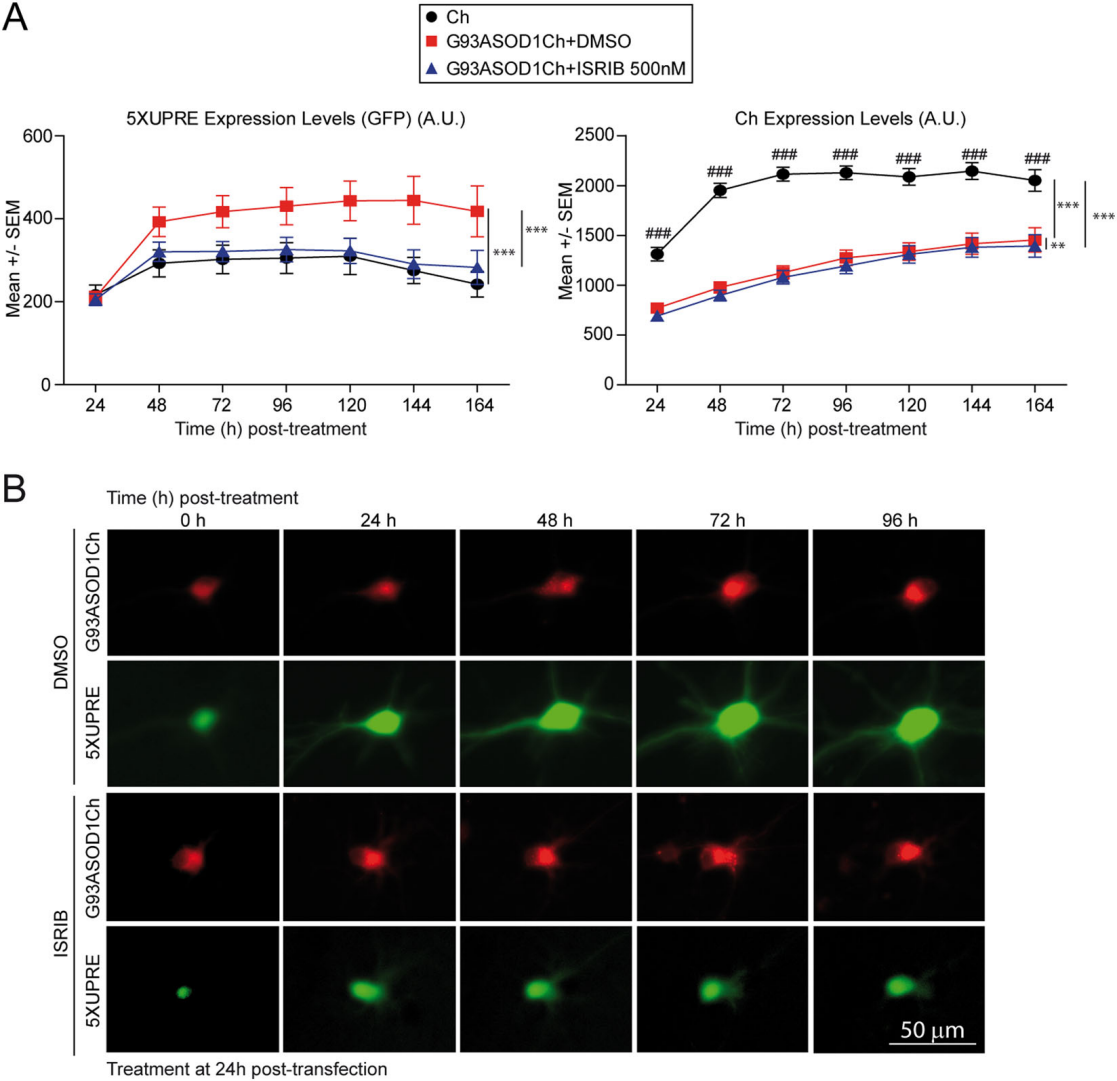
ISRIB decreases G93A SOD1-dependent ER stress signaling. **a** Left graph shows GFP fluorescent signal from the 5XUPRE reporter measured longitudinally in individual neurons expressing G93ASOD1Ch or Ch (control) and treated with DMSO and 500 nM ISRIB. Right graph shows the quantification of Ch fluorescence over the experimental time as surrogate of mutant SOD1 and Ch protein levels from the neurons analyzed. Pooled data from three independent experiments (~100 neurons tracked per condition in each experiment), two-way ANOVA and Bonferroni multiple comparisons; 5XUPRE levels change significantly among conditions (*F*_2,3799_ = 22.9, *p* < 0.001) and with time (*F*_6,3799_ = 6.6, *p* < 0.001). Right graph: Quantification of Ch levels from neurons on the left. Protein levels change significantly among conditions (*F*_2,3799_ = 1037, *p* < 0.001) and with time (*F*_6,3799_ = 135.8, *p* < 0.001). There is significant interaction in the conditions with time (*F*_12,3799_ = 6.9, *p*_int_ < 0.001). Significant differences of treatments: **p < 0.01, ***p < 0.001. Significant differences among time: ^###^*p* < 0.001. Error bars indicate standard error of the mean (SEM). **b** Representative images of longitudinal monitoring of UPR activation over time in single living neurons co-transfected with 5XUPRE transcriptional reporter and G93ASOD1Ch and treated with DMSO or ISRIB (500 nM). (Upper row) Red fluorescence intensity as a surrogate of G93ASOD1Ch expression levels. (Lower row) Green fluorescence intensity documents UPR activation.

The fine-tuning of PERK signaling by ISRIB could reduce the neurotoxic translational effects of PERK while keeping up its neuroprotective output. Other than that, blunting PERK may enhance signaling from parallel, independent UPR mechanisms. To determine to which extent ISRIB modulated IRE1 activation in G93A SOD1-expressing neurons, we monitored UPRE-dependent transcription activation longitudinally in individual neurons in the presence of ISRIB. Protein levels of G93ASOD1Ch and Ch were simultaneously monitored. As ISRIB relieves translation inhibition in neurons, we anticipated higher G93A SOD1 levels in ISRIB-treated neurons. However, only a minor decrease (5% less than in vehicle-treated cultures) of G93A SOD1 protein levels was observed in ISRIB-treated neurons (Fig. 6a). Surprisingly, ISRIB reduced 5XUPRE fluorescence levels in G93A SOD1-expressing neurons to control, Ch levels (Fig. 6a, b). This finding suggest that the unique modulation of PERK signaling by ISRIB improves neuronal survival in a mutant SOD1-based model of fALS, through a predominant mechanism whereby ER stress is alleviated.

## Discussion

One of the main challenges in the study of neurodegenerative disorders is the identification of molecular mechanisms that contribute to neuronal death or survival, to then develop therapeutic strategies aimed to suppress or enhance them, respectively. In ALS, different attempts have been conducted to establish the role of specific UPR signaling events/components, yielding conflicting and sometimes opposing conclusions. To address this controversy, we have developed a neuronal model of neurodegeneration based on the expression of the G93A SOD1 mutant where neuronal survival and UPR activation were simultaneously monitored by longitudinal survival analysis. Even if SOD1 is not an ER luminal protein itself, mutant SOD1 is one of the best genetic models of neurodegeneration linking UPR activation and disease progression^26,30–32^. Beyond a quantitative confirmation of UPR activation, our experimental system served to demonstrate that indeed UPR activation precedes and is associated with neuronal death. The link between UPR and ALS neurodegeneration is specific, since it was not observed in other models of neurodegeneration (i.e., E46K aSyn mutant^45^).

Pharmacological modulation of IRE1 and PERK signaling identified the ISR as a therapeutic target for mutant SOD1-induced ALS. In contrast, inhibiting IRE1 RNAse activity did not affect survival of G93A SOD1-expressing neurons. PERK/ISR inhibition has been found to relieve neurotoxicity in animal and cellular models of ALS (TDP43 and C9orf72) or other pathologies, such as prionic disease or vanishing white matter disease^29,42,60,61^. These evidences indicate that tuning translation conveys robustness to neurons in pathologies where ER protein folding homeostasis is disrupted.

Importantly, ISRIB (but not GSK) improved survival of neurons expressing mutant SOD1. To date, we cannot rule out the possibility that eIF2α kinases other than PERK—that would be counteracted by ISRIB, but not by GSK—contribute to ISR in ALS. Yet, under ER stress conditions the downstream modulation of PERK by ISRIB conveys a qualitatively distinct output in neurons. Based on our finding, we propose a model whereby ISRIB uncouples pro-survival and neurotoxic capacities of PERK signaling (Supplementary Fig. S7); ISRIB would relieve the neurotoxic translational repression imposed by PERK while maintaining translation of uORF-containing mRNAs (i.e., ATF4), thereby fostering neuronal survival. Beyond the pharmacological mechanism whereby ISRIB achieves this regulation, our findings identify the neuroprotective contribution of uORF-dependent translation in mutant SOD1 neurotoxicity, and highlight the relevance of PERK modulation. In line with our findings, partial restoration of translation by ISRIB was found to prevent neurodegeneration in a murine prion disease model^60^.

In our experimental model, exacerbation of PERK signaling by Sephin1 did not impede neuronal death. However, it is conceivable that—within a specific activation range—PERK stimulation could balance its neuroprotective or neurotoxic capacities and promote neuronal survival of mutant SOD1-expressing neurons. If that was the case, different “proteostatic” solutions could be approached to treat ALS.

Even when the mechanism of action of ISRIB has been well characterized^62,63^, we do not understand why its inhibitory capacities are limited in neurons. Recent functional and structural studies hint plausible explanations for this phenomenon: Rabouw et al.^64^ demonstrated that ISRIB antagonizes the ISR only when p-eIF2α levels are below a critical threshold. On the other hand, the phosphorylation status of neuronal eIF2B subunits (the molecular target of ISRIB) may be modulated in response to specific cues, thereby driving changes in translation initiation^65^. As a final, exciting possibility, neurospecific metabolites could occupy the natural cavity within eIF2B where ISRIB binds, thereby limiting the extent of ISRIB action^62^.

As the capacity of UPR to cope with ER stress relies on the coordinated action of its three independent branches, PERK inhibition should promote the compensatory activation of the IRE1 and ATF6 pathways. Surprisingly, in mutant SOD1-expressing neurons, ISRIB brought UPR transcription back to basal levels. Given the tight causal relationship between UPR and neuronal death in ALS, we postulate that ISRIB promotes survival by alleviating mutant SOD1-induced ER stress. The fine modulation of ISRIB may overcome pathological mechanisms by which mutant SOD1 impairs neuronal function like inhibiting ER-associated degradation^66^, disrupting protein clearance pathways^67^, or altering axonal transport^17,68^.

In summary, ISRIB-mediated translational modulation provides a solution to the toxicity imposed by mutant SOD1. These findings could be now expanded to other fALS models and adapted as a valuable methodology to study the contribution of UPR to neurodegeneration. Understanding the pathological processes that ISRIB translational reprogramming overcomes will serve to design new/better ALS therapeutic strategies.

## Supplementary information

Supplementary Figure Legends

Figure S1

Figure S2

Figure S3

Figure S4

Figure S5

Figure S6

Figure S7
