# Supplementary material for: Fine tuning of the unfolded protein response by ISRIB improves neuronal survival in a model of amyotrophic lateral sclerosis
Source: Cell Death Dis. 2020 May 26;11(5):397. doi: 10.1038/s41419-020-2601-2 (PMC7250913; doi:10.1038/s41419-020-2601-2)
Supplement: Supplementary file 1 — Supplementary Figure Legends [file 41419_2020_2601_MOESM1_ESM.docx]

**Supplementary Figure Legends**

**Figure S1.** Example of longitudinal tracking of individual primary cortical neurons expressing monomeric Cherry (Ch) and Ch-tagged SOD1 versions (WT and mutants G85R and G93A) with automated microscopy. Red arrows indicate neurons that die over the course of the experiment. Green arrows point to neurons tracked longitudinally that survive.

**Figure S2. Characterization of SOD1 Ch-tagged versions.** A) Fluorescence intensity of SOD1 Ch-tagged constructs is a faithful surrogate of SOD1 protein expression levels. Rat primary cortical neurons transiently transfected with mCherry (Ch) and (Ch)-tagged versions of SOD1 (SOD1Ch, G85RSOD1Ch, G93ASOD1Ch) were immunostained with a specific antibody against Ch (anti-Ch). Fluorescence intensity from Ch and from secondary antibodies recognizing anti-Ch was analyzed. Correlation coefficient (r) and *P* values were estimated by Spearman correlation analysis (A.U., arbitrary units). B) Same experiment as in (A) but using a specific antibody against SOD1 (pan-SOD1). C) Rat cortical neurons were co-transfected with GFP and different versions of human SOD1 (WT/G85R/G93A) and empty vector (e.v.) as control. Neurons were immunostained with SOD1 specific antibodies (pan-SOD1 and conformational C4F6, ^1-3^). The fluorescence intensity of GFP and of the secondary antibodies recognizing pan-SOD1 and C4F6 was quantified in individual neurons. The graphs show SOD1 antibody binding (pan-SOD1 or C4F6) normalized by GFP in individual neurons (Number of neurons per condition: Left: GFP+e.v.=20, GFP+SOD1WT=20, GFP+G85RSOD1=17, GFP+G93ASOD1=19; Right: GFP+e.v.=25, GFP+SOD1WT=25, GFP+G85RSOD1=25, GFP+G93ASOD1=26; Kruskal-Wallis and Dunn's post-hoc test). D) Same experiment and quantification as in B but in primary neurons transiently transfected with Ch-tagged versions of SOD1. The graphs show SOD1 antibody binding (pan-SOD1 or C4F6) normalized by Ch in individual neurons (Number of neurons per condition: Left: GFP+e.v.=17, GFP+SOD1WT=19, GFP+G85RSOD1=21, GFP+G93ASOD1=19; Right: GFP+e.v.=25, GFP+SOD1WT=26, GFP+G85RSOD1=25, GFP+G93ASOD1=26; Kruskal-Wallis and Dunn's post-hoc test). *p<0.05, ***p<0.001). E) Neurons were co-transfected with plasmids expressing GFP and human SOD1 (WT/G85R/G93A) and immunostained with a pan anti-SOD1 antibody. Fluorescence intensity from GFP and secondary antibodies recognizing pan anti-SOD1 was quantified in individual neurons. Every GFP positive neuron expressed SOD1 protein compared to control conditions (GFP co-transfected with empty vector (e.v.)) (20 neurons per condition). Thus, GFP fluorescence in neurons is a good surrogate of the expression of untagged SOD1 versions.

**Figure S3.**

*Rationale for Supplementary Figure 3:*

*We tested the capacity of a transcriptional UPR reporter bearing five tandem copies of the unfolded protein response element (5XUPRE) (Fig. 2A) to document ER stress responses in cells. HEK293 cells were co-transfected with plasmids expressing the 5XUPRE-GFP reporter and Ch (Ch was expressed from a constitutive promoter to track transfected cells). Twenty-four hours after transfection cells were treated with the SERCA pump inhibitor thapsigargin (Thap), which induces acute ER stress, and imaged for a period of 48 hours. As anticipated, a burst of 5XUPRE-derived GFP fluorescence was scored (Fig. S3A). In addition to 5XUPRE, we generated a second UPR reporter, SpR, based on the non-canonical splicing of XBP1. Under ER stress, removal of a 26-nucleotide intron within the XBP1 coding sequence provides a translational frameshift that allows the translation of the potent transcription factor, XBP1s. In a similar fashion to other UPR splicing-based reporters, the GFP coding sequence was placed downstream the XBP1 intron, such that it is only in frame after splicing (Fig. 2D). HeLa cells were co-transfected with plasmids expressing Ch and SpR or empty vector (e.v.) as control, treated with Thap 1μM and imaged by automated microscopy over 24 hours. The ratio of GFP over Ch was quantified in individual cells as an indicator of XBP1 splicing-dependent UPR activation. SpR monitored efficiently UPR activation (Fig. S3B, S3C).*

*To further confirm that the fluorescence derived from the 5XUPRE reporter depends on the five tandem UPRE repeats, we removed them from the reporter to create a new control plasmid (pGL3-GFP). When tested in neurons, the levels of fluorescence derived from the expression of pGL3-GFP reporter were remarkably lower than the ones produced by the 5XUPRE reporter (Fig. S3D). Thus, levels of basal 5XUPRE fluorescence are clearly above the background fluorescence of our primary neuronal cultures. Next, we tested the performance of 5XUPRE reporter or pGL3-GFP when transfected into neurons upon Tun 0.625µg/ml treatment. In Tun-treated neurons, fluorescence levels increase in a time-dependent manner in neurons transfected with 5XUPRE, but not in pGL3-GFP-transfected neurons (Fig. S3D), indicating that the ER stress increases reporter transcription in a UPRE-dependent manner. Finally, we confirmed the specificity of SpR activation upon ER induced stress with Tun in primary neurons. SpR-dependent fluorescence was a bona fide consequence of UPR splicing, since pharmacological inhibition of the IRE1 endonuclease domain by 4μ8C kept reporter fluorescence at basal levels (Fig. S3E).*

*Once validated, we used these reporters to document UPR activation in the course of mutant SOD1-dependent neurodegeneration.*

**Figure S3. Characterization of 5XUPRE and SpR fluorescent reporters.** A) HEK293 cells were co-transfected with Ch and 5XUPRE GFP (Ch and empty vector (e.v.) as control). ER stress was induced with Thapsigargin (Thap). Co-transfected cells were imaged at 0, 4, 24 and 48 hours (h) after 1μM Thap addition (10 non-overlapping random fields per condition). The graph shows the quantification of UPR activation at each time point (ratio GFP/Ch signal measured on individual cells and normalized by the mean value at 0h) (number of cells per condition at 0h, 4h, 24h and 48h: Ch+e.v.=299, 468, 719, 920; Ch+5XUPRE GFP=482, 616, 1172, 1202). B) Images of HeLa cells co-transfected with plasmids expressing Ch and SpR or empty vector (e.v.) as control. Co-transfected cells were imaged (10 non-overlapping random fields per condition) at 0, 8 and 24 hours (h) after 1 μM Thap addition. The graph shows the quantification of UPR activation at each time point (ratio GFP/Ch signal measured on individual cells). SpR values were compared to control conditions (e.v.) (number of cells per condition at 0h, 8h and 24h: e.v.=127, 149, 160; SpR=270, 275, 361). Kruskal-Wallis and Dunn's post-hoc test. C) Protein extracts of co-transfected HeLa cells with plasmids expressing Ch and SpR or empty vector (e.v.) were prepared at 0, 4 and 8h after treatment with 1 μM Thap. WB analysis was performed against GFP and GADPH (loading control). D) Images of co-transfected primary cortical neurons with Ch and pGL3-GFP or 5XUPRE plasmids untreated or 48h after treatment with Tun 0.625 μg/mL. Graph showing the quantification of UPR activation at 0h, 8h, 24h and 48 h after Tun addition in individual neurons (ratio GFP/Ch signal). Values of Ch + 5XUPRE GFP neurons at 24h and 48h were compared with values at 0h (number of neurons per condition at 0h, 8h, 24h and 48h: Ch + pGL3-GFP= 498, 495, 356, 141; Ch + 5XUPRE-GFP= 572, 525, 366, 124). Kruskal-Wallis and Dunn's post-hoc test. Error bars indicate 95% confidence intervals (CIs); ***p<0.001. E) Images of co-transfected primary cortical neurons with Ch and SpR plasmid at 0h and 8h after treatment with 0.625 μg/mL Tunicamycin (Tun), 16 μM 4μ8C, and DMSO (control). Graph showing the quantification of UPR activation at each time point on individual neurons (ratio GFP/Ch signal). Tun+4μ8C versus Tun values were compared (number of neurons per condition at 0h, 4h and 8h: DMSO=87, 65, 109; Tun=46, 44, 49: Tun+4μ8C=48, 36, 17). Kruskal-Wallis and Dunn's post-hoc test. Error bars indicate 95% confidence intervals (CIs); *p<0.05, **p<0.01, ***p<0.001.

**Figure S4. Analysis of nuclear ATF4 proteins levels in neurons under acute ER stress.** Rat primary cortical neurons were treated with 100nM Thapsigargin (Thap) for 6h. Nuclear ATF4 protein levels in neurons (MAP2+ cells) and astrocytes (GFAP+ cells) were analyzed by immunofluorescence experiments with specific antibodies. A) Images showing nuclear ATF4 levels in neurons (MAP2+ cells) after Thap addition. Quantification of nuclear fluorescence intensity (ATF4 levels) in neuronal and non neuronal cells (MAP2+ and MAP2- cells). Thap addition efficiently induces an increase of ATF4 nuclear levels in neuronal and non-neuronal cells. Number of neurons per condition: DMSO=45 and Thap=85, number of non-neuronal cells per condition: DMSO=89 and Thap=87. Mann Whitney test. B) Images showing nuclear ATF4 levels in astrocytes (GFAP+ cells) after Thap addition. Quantification of nuclear fluorescence intensity (ATF4 levels) in neurons (MAP2+ cells) and astrocytes (GFAP+ cells). Thap addition increases ATF4 nuclear levels in neurons and astrocytes. Number of neurons per condition: DMSO=71 and Thap=37, number of astrocytres per condition: DMSO=37 and Thap=38. Mann Whitney test. Error bars indicate 95% confidence intervals (CIs), ***p<0.001. **Analysis of ATF4 proteins levels in G93A SOD1 expressing neurons and HEK293 cells.** C) Rat cortical neurons were co-transfected with GFP and human SOD1 versions (WT/G93A) or empty vector (e.v.) as control. Neurons were immunostained with ATF4 specific antibody at 5 days post-transfection. The graph shows the correlation between GFP and ATF4 levels for the different neuronal groups (Spearman r coefficient for each group e.v.=0.78 ***p<0.001, SOD1 WT=0.78 ***p<0.001, SOD1 G93A=0.70 ***p<0.001). G93A SOD1 expressing neurons show higher ATF4 levels despite having lower GFP (number of neurons: e.v.=26, SOD1 WT=25, SOD1 G93A=26). D) HEK293 cells were transiently transfected with expression plasmids of SOD1 (WT and pathological mutants G85R and G93A) and empty vector (e.v.) as control. Protein levels of ATF4 and XBP1 were analyzed by Western blots (GADPH as loading control).

**Figure S5. ISRIB and GSK decrease ATF4 levels of Thapsigargin (Thap)-treated HEK293 cells.** Quantification and statistical analysis of ATF4 and XBP1s levels to determine the effect of 500nM ISRIB, 500nM GSK and 50μM 4μ8C (treatments) in HEK293 cells treated with 100nM Thap at 1.5 and 3 hours (time). Data analyzed by two-way ANOVA followed by Bonferroni pairwise comparisons; n=3 independent experiments represented as the mean +/- standard deviation (SD). A) ATF4 levels change significantly among the different treatments (F_3,16_=39.2, p<0.001). There is a significant interaction in the treatments with time (F_3,16_=3.6 p_int_<0.05). B) XBP1 levels change significantly with time (F_1,16_=15.9, p<0.01) and among treatments (F_3,16_=7.6, p<0.01). No interaction was detected. 4μ8C is the only inhibitor that reduces XBP1s levels. Significant differences of treatments: ***p < 0.001, **p < 0.01. Significant differences between time: ##p < 0.01.

**Figure S6. Sephin1 does not improve survival of G93A SOD1 expressing neurons.** A) Western blot analysis of ATF4, XBP1s and GAPDH (loading control) from Hela protein extracts treated with 100nM Thapsigargin (Thap) and 50 μM 4μ8C, 500nM GSK, 10 μM Sephin1, and DMSO as control. B) Cumulative hazard estimates of primary neurons expressing SOD1 pathological version G93ASOD1Ch and treated with Sephin1 10 μM (Ch as control). CPH analysis; pooled data from 2 independent experiments, n=number of neurons, * p<0.05.

**Figure S7**. A) Table summarizing the effects of UPR modulating drugs in neuronal survival (in the G93A SOD1 model) and in their capacity to respond to chemically induce ER stress. B) Model to explain the potential neuroprotective mechanism of ISRIB in G93A SOD1-expressing neurons. ER stress induced by mutant SOD1 elicits eIF2α phosphorylation by PERK. In turn, phospho-eIF2α regulates translation in two different modes: It dampens translation of most mRNA, but selectively enhances the translation of transcripts containing small ORFs at their 5’ UTRs (uORF). Our results favor a model where translation of uORF-containing stress-related proteins encodes a pro-survival coping response to mutant SOD1 neurotoxicity, while the reduction of protein synthesis contributes to neurodegeneration. C) ISRIB remodels neuronal translation in a unique manner, increasing general translation rates without inhibiting the expression of uORF-containing mRNAs. ISRIB-mediated translational reprogramming leads to a reduction in UPR signaling that may result from the alleviation of ER stress. NT, non tested.

**References**

1 Brotherton, T. E. *et al.* Localization of a toxic form of superoxide dismutase 1 protein to pathologically affected tissues in familial ALS. *Proc Natl Acad Sci U S A* 109, 5505-5510, doi:1115009109 [pii]

10.1073/pnas.1115009109 (2012).

2 Urushitani, M., Ezzi, S. A. & Julien, J. P. Therapeutic effects of immunization with mutant superoxide dismutase in mice models of amyotrophic lateral sclerosis. *Proc Natl Acad Sci U S A* 104, 2495-2500, doi:0606201104 [pii]

10.1073/pnas.0606201104 (2007).

3 Rotunno, M. S. *et al.* Identification of a misfolded region in superoxide dismutase 1 that is exposed in amyotrophic lateral sclerosis. *J Biol Chem* 289, 28527-28538, doi:M114.581801 [pii]

10.1074/jbc.M114.581801 (2014).
