# Supplementary figures and images for: Fine tuning of the unfolded protein response by ISRIB improves neuronal survival in a model of amyotrophic lateral sclerosis
Source: Cell Death Dis. 2020 May 26;11(5):397. doi: 10.1038/s41419-020-2601-2 (PMC7250913; doi:10.1038/s41419-020-2601-2)

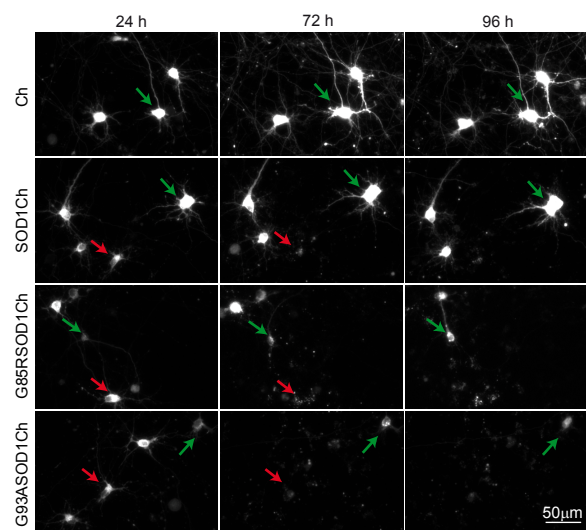

Supplement: Supplementary file 2 — Figure S1 [file 41419_2020_2601_MOESM2_ESM.pdf]

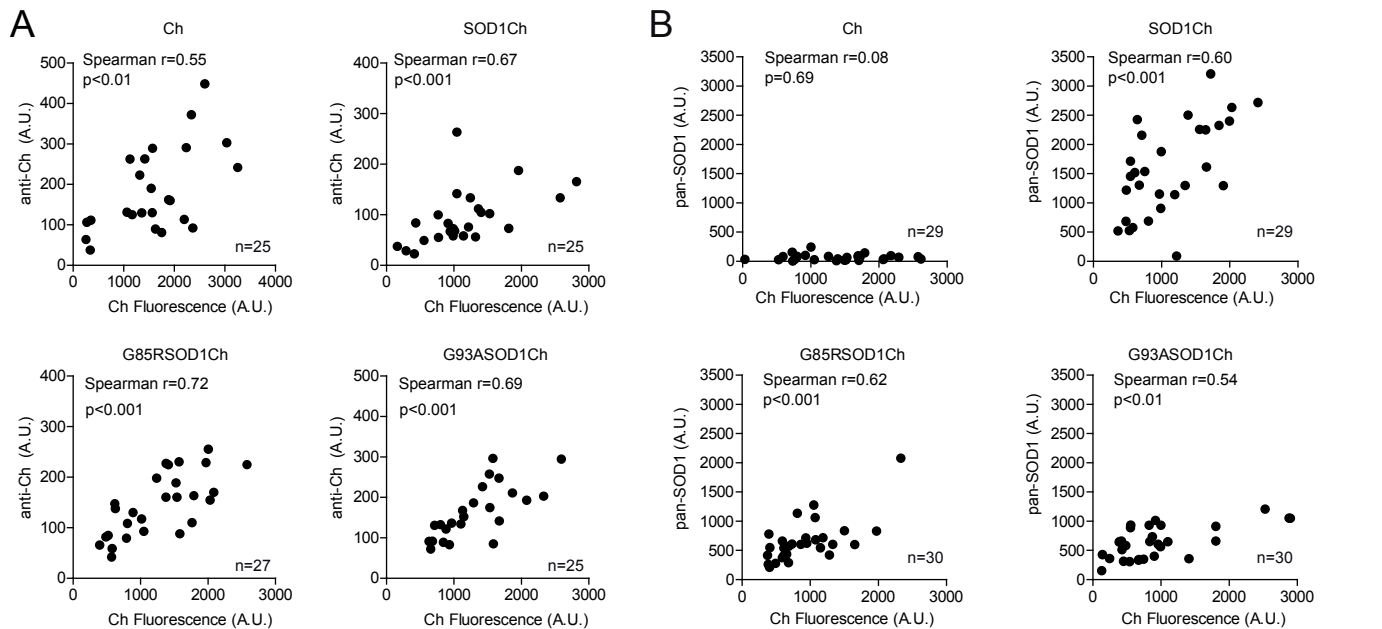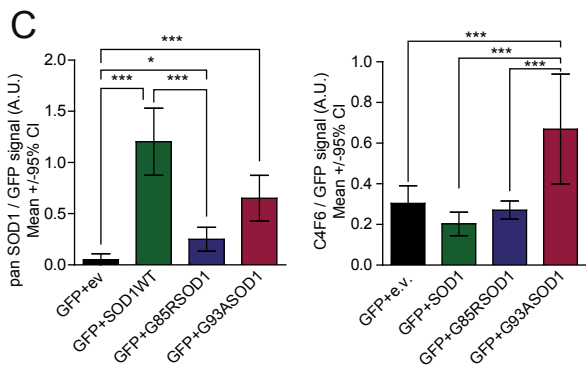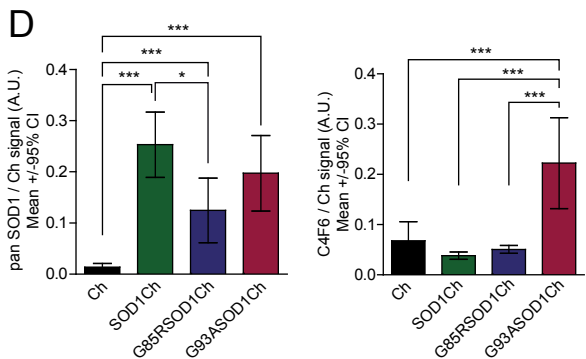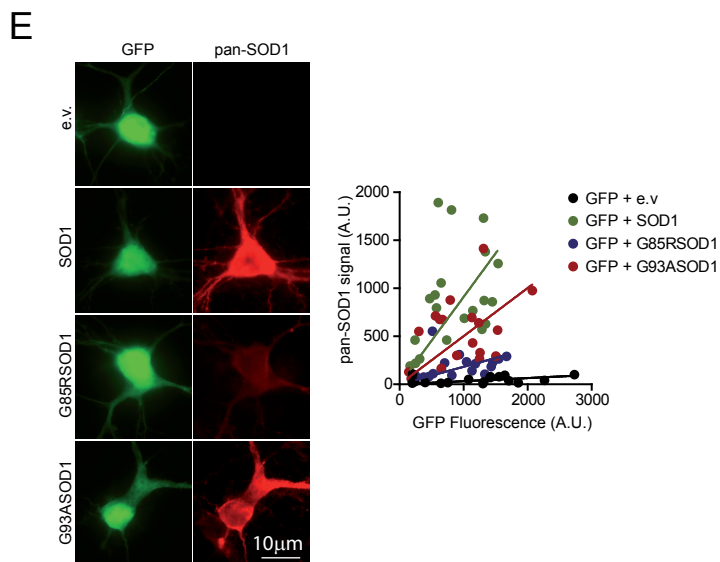

Supplement: Supplementary file 3 — Figure S2 [file 41419_2020_2601_MOESM3_ESM.pdf]

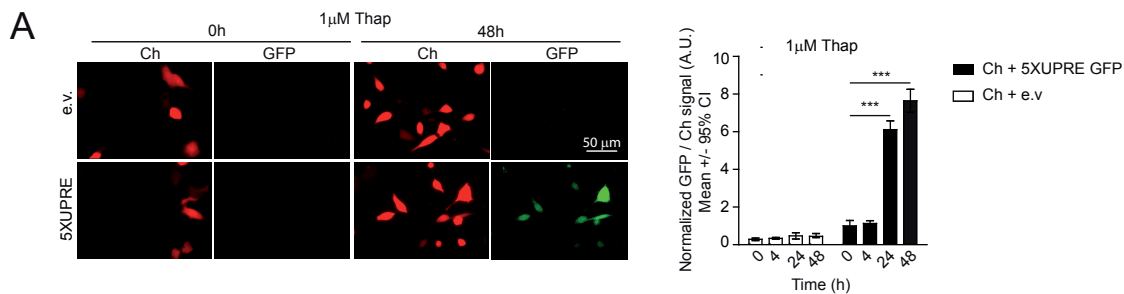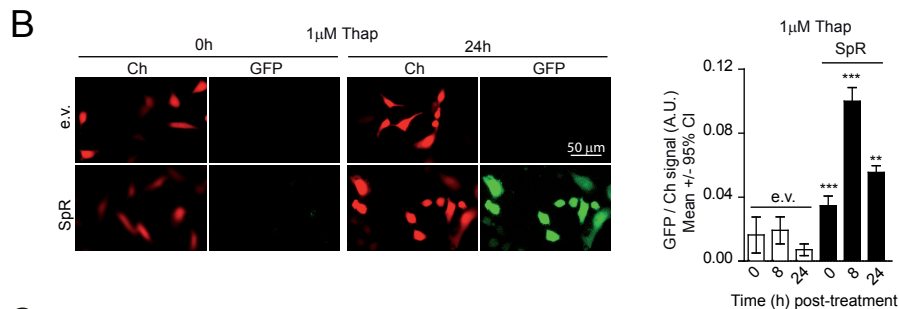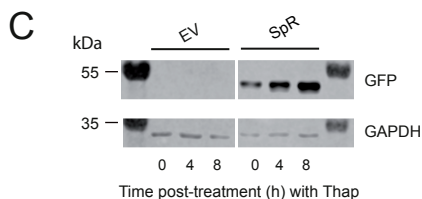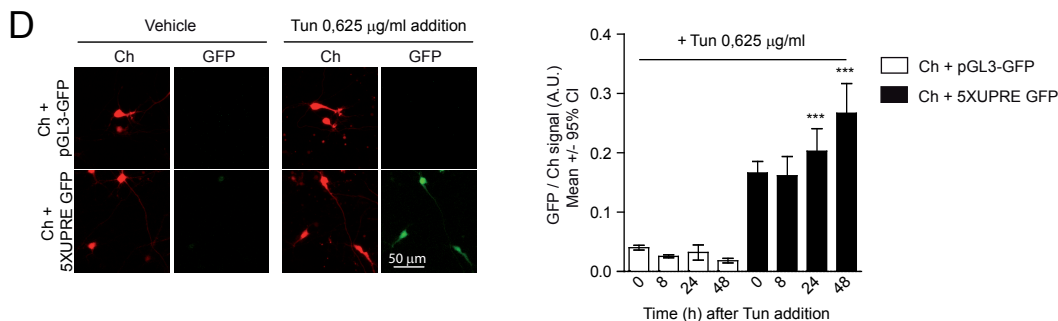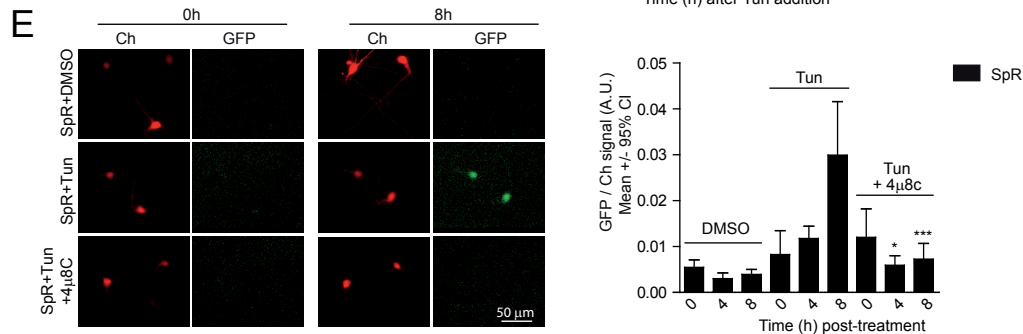

Supplement: Supplementary file 4 — Figure S3 [file 41419_2020_2601_MOESM4_ESM.pdf]

**A**

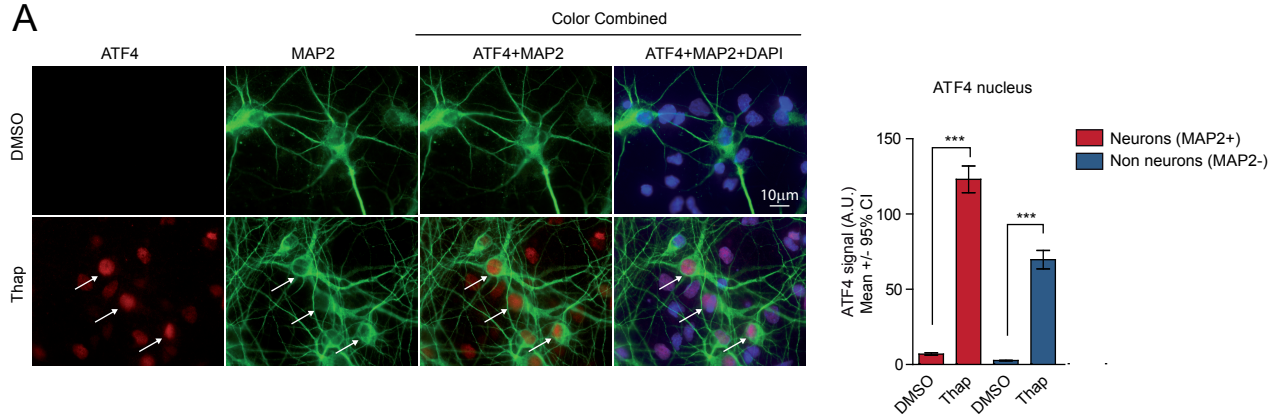

**B**

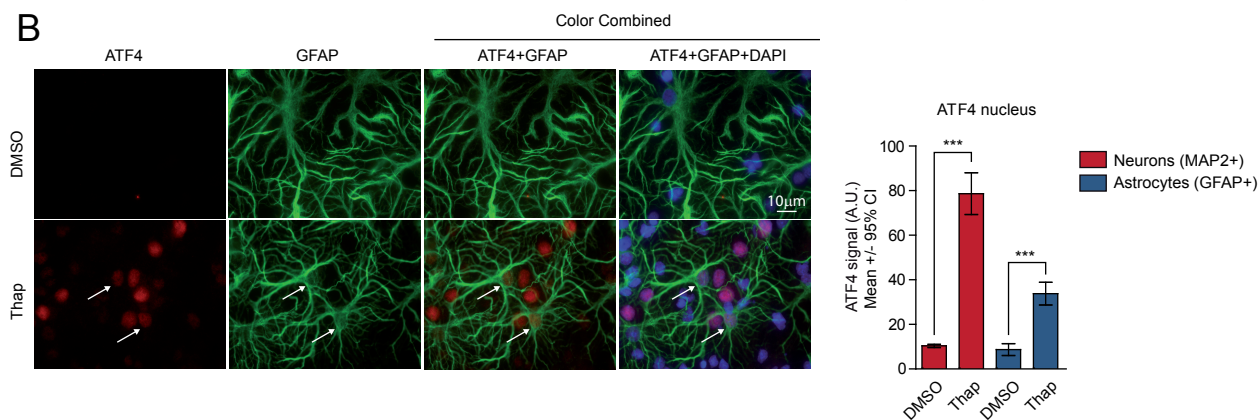

**C**

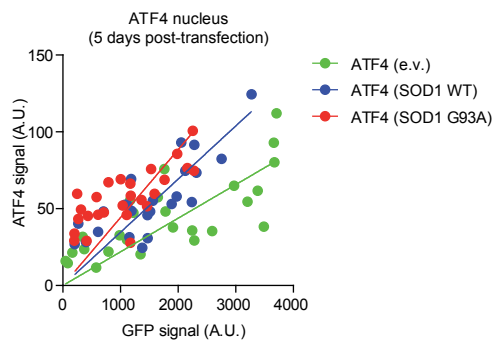

**D**

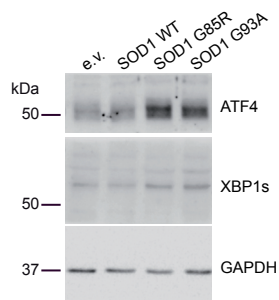

Supplement: Supplementary file 5 — Figure S4 [file 41419_2020_2601_MOESM5_ESM.pdf]

**A**

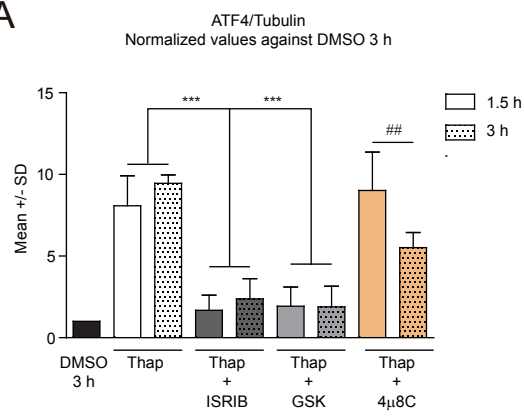

**B**

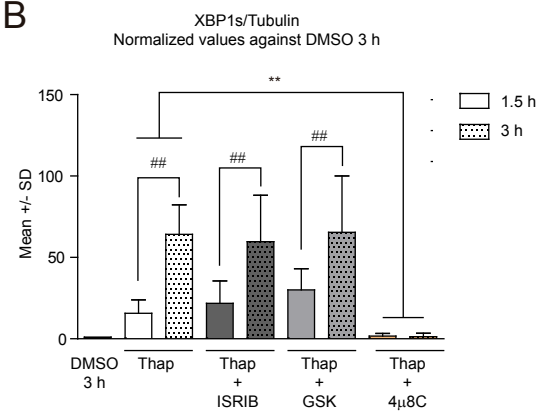

Supplement: Supplementary file 6 — Figure S5 [file 41419_2020_2601_MOESM6_ESM.pdf]

A

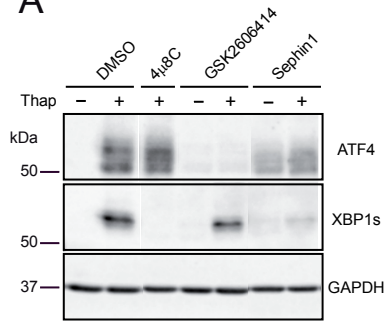

B

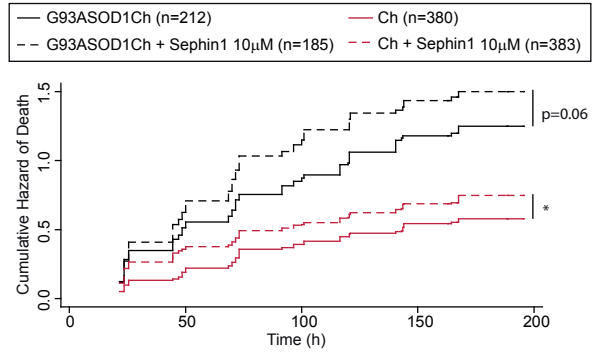

Supplement: Supplementary file 7 — Figure S6 [file 41419_2020_2601_MOESM7_ESM.pdf]
