# Supplementary material for: Fine tuning of the unfolded protein response by ISRIB improves neuronal survival in a model of amyotrophic lateral sclerosis
Source: Cell Death Dis. 2020 May 26;11(5):397. doi: 10.1038/s41419-020-2601-2 (PMC7250913; doi:10.1038/s41419-020-2601-2)

A

Response to ER stress
Neurons / HEK293

Compound	Molecular Target	Improves Survival	ATF4 Translation (Neurons)	ATF4 Translation (HEK293)	Protein Synthesis (Neurons)	Protein Synthesis (HEK293)
ISRIB	eIF2B dimerization enhancer	Yes	↓	↓↓↓	↑↑	↑↑
GSK2606414	PERK kinase domain	No	↓↓↓	↓↓↓	↑↑	↑↑
4μ8C	IRE1α RNAse domain	No	NT	NT	NT	NT

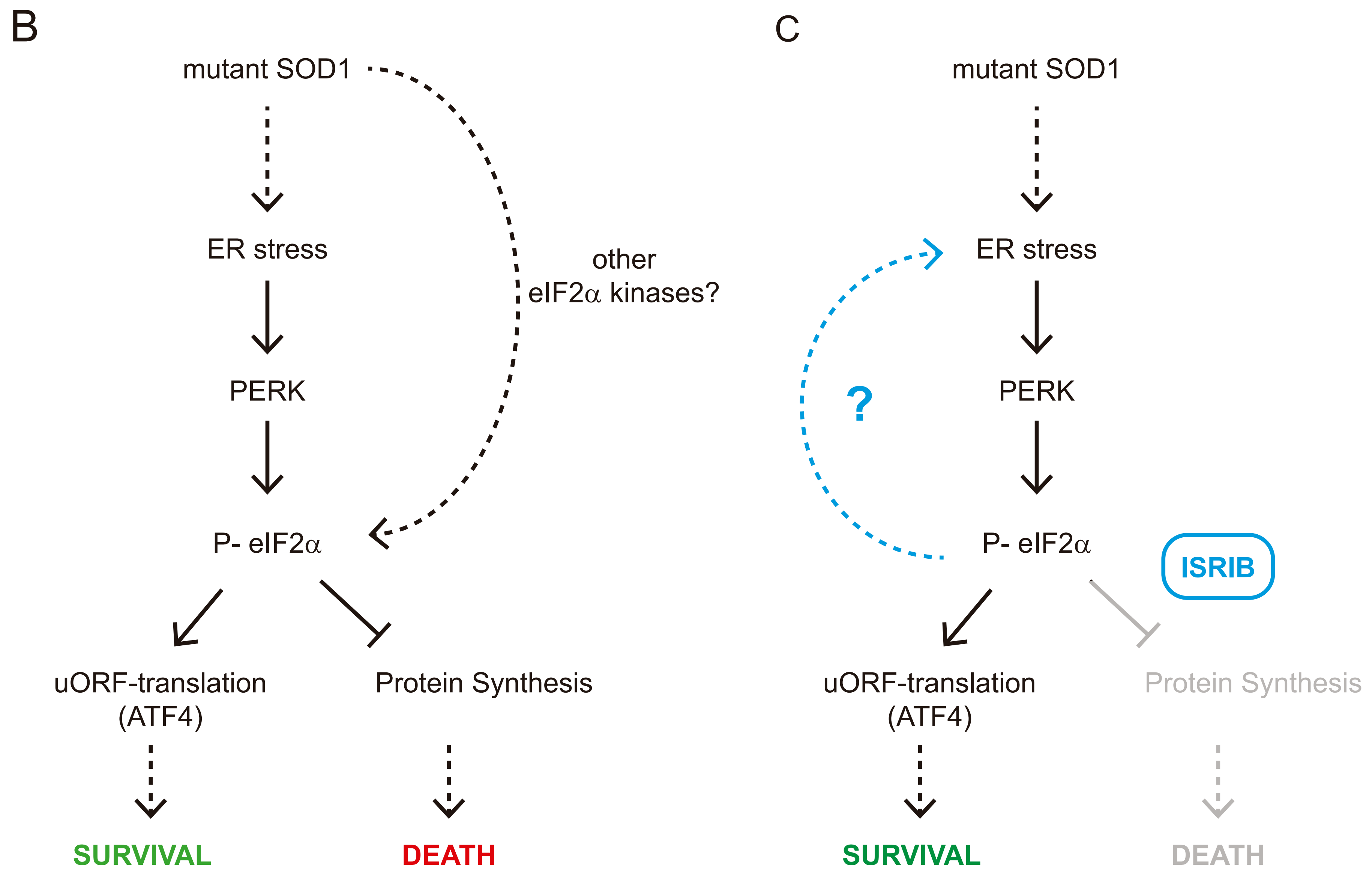

Supplement: Supplementary file 8 — Figure S7 [file 41419_2020_2601_MOESM8_ESM.pdf]
